# Targeting mitochondrial bioenergetics: the “Achilles’ heel” of *Leishmania*

**DOI:** 10.1186/s13071-026-07247-x

**Published:** 2026-03-09

**Authors:** Deblina Sarkar, Sritama De Sarkar, Deep Goswami, Mitali Chatterjee

**Affiliations:** Department of Pharmacology, Institute of Post Graduate Medical Education and Research (IPGMER), 244B Acharya JC Bose Road, Kolkata, West Bengal 700 020 India

**Keywords:** Amphotericin B, Antileishmanials, Bioenergetics, Hexadecylphosphocholine (HePC), Leishmaniasis, Metabolic reprogramming, Mitochondria

## Abstract

**Background:**

The parasite *Leishmania* is the causative agent of leishmaniasis and relies on a single “mitochondrion” as its “powerhouse”. It also has a compromised antioxidant defense system. Consequently, potential therapeutic strategies include triggering mitochondrial dysfunction along with subversion of host metabolic bioenergetics; however, such information remains poorly defined. The focus of this study was to delineate the impact of antileishmanials amphotericin B (Ampho B) and miltefosine (hexadecylphosphocholine [HePC]) on the metabolic bioenergetics of *Leishmania* parasites vis-à-vis mammalian macrophages.

**Methods:**

In promastigotes, the redox status was evaluated by flow cytometry; levels of ATP were measured by chemiluminescence; and oxygen consumption rate (OCR), extracellular acidification rate (ECAR), and substrate utilization were assessed by XFp Analyzer. In *Leishmania donovani* (*L. donovani*)-infected macrophages, expression of metabolic bioenergetics related regulatory molecules was assessed by droplet digital polymerase chain reaction (ddPCR) and immunoblotting.

**Results:**

In promastigotes, at their respective 50% and 90% inhibitory concentrations (IC_50_ and IC_90_), Ampho B and HePC disrupted redox homeostasis, enhanced generation of mitochondrial superoxide, depleted OCR and ATP, and caused a greater degree of mitochondrial inhibition with HePC than with Ampho B. The *L. donovani* promastigotes sourced acetyl CoA primarily from the fatty acid oxidation pathway for mitochondrial tricarboxylic acid cycle, and inhibition of the mitochondrial fatty acid oxidation was higher with Ampho B. In *L. donovani*-infected macrophages, there was significantly increased expression of the *Ampk* axis (*Ampk*–*Lkb1*–*Sirt1*), mitochondrial biogenesis marker *Pgc1α*, and markers of oxidative phosphorylation (*Cox IV*, *Atp synthase*). The rate-limiting enzymes of glycolysis, namely  *HkII*, *Pfk*, *Pkm2*, and glucose transporter (*Glut1*), were enhanced, but expression of *Mtor* was decreased. All markers of the *Ampk* axis and oxidative phosphorylation were significantly curtailed by both antileishmanials in favor of parasite clearance, whereas glycolytic markers remained unchanged; overall, the dampening of metabolic bioenergetics by HePC was greater than that caused by Ampho B.

**Conclusions:**

Targeting the “mitochondrion” and metabolic reprogramming are effective leishmanicidal strategies adopted by HePC and Ampho B, with the degree of inhibition by HePC exceeding that of Ampho B. Accordingly, screening for compounds capable of mediating metabolic reprogramming could augment the limited armamentarium of antileishmanials.

**Graphical abstract:**

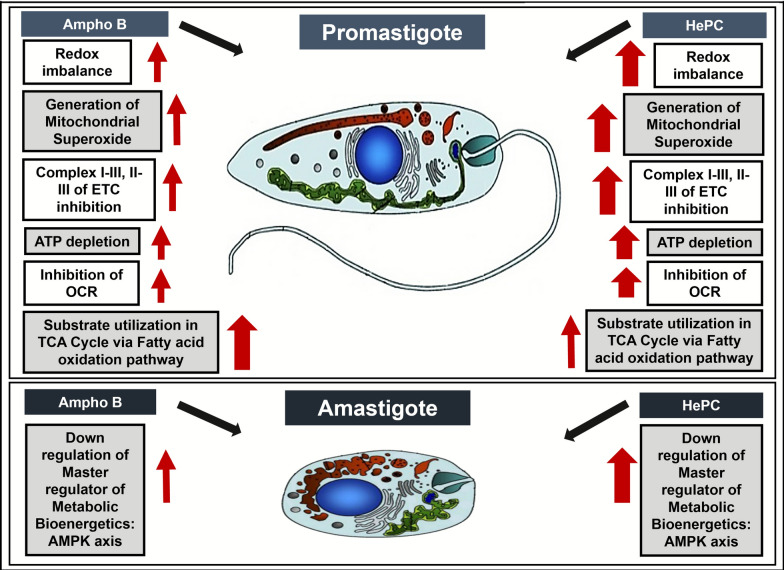

**Supplementary Information:**

The online version contains supplementary material available at 10.1186/s13071-026-07247-x.

## Background

Leishmaniasis, a neglected tropical disease (NTD), is the second most deadly parasitic disease after malaria, prevalent in > 98 countries that are mainly within tropical and subtropical areas [1]. Worldwide, nearly 15 million people are infected, and annually, 1.5–2 million new cases are identified, with fatality occurring in 30,000–70,000 individuals [2, 3]. The etiological agent of leishmaniasis is *Leishmania*, a unicellular digenetic protozoan parasite, which is transmitted by female *Phlebotomus* (Old World) and *Lutzomyia* (New World) sand flies. *Leishmania* alternates between promastigotes (the motile form) residing in sand flies and amastigotes (the nonmotile form) residing within host mammalian macrophages. There are more than 20 diverse species, causing a wide range of clinical manifestations that can be broadly differentiated into four major categories, namely cutaneous leishmaniasis (CL), mucocutaneous leishmaniasis (MCL), visceral leishmaniasis (VL), and post-kala-azar dermal leishmaniasis (PKDL) [1, 3].

The treatment of leishmaniasis remains a global challenge, as vaccines are not commercially available and treatment hinges entirely on chemotherapy. The available chemotherapy is limited, as it includes pentavalent antimonials, amphotericin B, miltefosine, and paromomycin, all of which are plagued with severe limitations such as toxicity, high production cost, decreased efficacy, difficulty in administration, and most importantly, the emergence of resistance and treatment failure [2, 4]. Hence, global efforts to develop alternative strategies for novel immunotherapeutic therapies for leishmaniasis are the need of the hour.

*Leishmania* parasites belong to the family Trypanosomatidae, order Kinetoplastida, and have a single “mitochondrion”, vis-à-vis mammalian cells that have numerous mitochondria [5]. Kinetoplastids possess exclusive organelles and metabolic pathways that are needed for growth and proliferation within hosts during various stages of their life cycle [5]. The “mitochondrion” is a crucial organelle for survival of *Leishmania*, as it exerts a vital role in regulation of essential metabolic pathways, generating ATP and mediating an apoptotic cell death, collectively emphasizing that the ''mitochondrion'' of *Leishmania* parasites could be the “Achilles’ heel” worthy of being targeted for leishmanicidal activity. Additionally, *Leishmania* parasites are vulnerable to oxidative stress as they lack catalase and the classical selenium-dependent glutathione peroxidase system [6]. Accordingly, mitochondria, being one of the major sources of reactive oxygen species (ROS), could be a promising molecular target for antileishmanial therapeutics.

An integral component of cell metabolism is their metabolic programming as it is needed for maintaining immune cell activation, differentiation, and survival [7, 8]. The cellular redox status and mitochondrial ROS (mtROS) are established key players that regulate the functioning of immune cells [7, 8]. Intracellular pathogens (*Salmonella* spp., *Mycobacterium tuberculosis*, viruses (e.g., hepatitis B virus, human papilloma virus), and parasites (e.g., *Plasmodium* spp., *Toxoplasma* spp.) modulate the host metabolic pathways, ensuring that the microenvironment is conducive for their propagation [8–10]. The metabolic reprogramming of macrophages by *Leishmania* parasites determines disease progression [9]. The M1 microbicidal proinflammatory macrophages can trigger an oxidative burst, which is associated with heightened glycolytic metabolism, possibly to rapidly generate ATP and ensure parasite elimination. In contrast, anti-inflammatory M2 macrophages facilitate intramacrophagic parasite survival/disease progression, favored by low production of nitric oxide (NO), enhanced mitochondrial oxidative phosphorylation, and tricarboxylic acid cycle (TCA) cycle functions (especially fatty acid metabolism) [8, 11]. Accordingly, the aim of this study was to evaluate the impact of conventional antileishmanials, amphotericin B (ampho B) and miltefosine (hexadecylphosphocholine [HePC]), on *L. donovani* promastigotes, focusing on their redox status and elucidating the mechanism(s) adopted by parasites for subversion of host metabolic bioenergetics following infection.

## Methods

### Reagents

All chemicals were of analytical grade and obtained from Sigma-Aldrich (St. Louis, MO, USA), except the following: 3-(4,5-dimethylthiazol-2-yl)-5-(3-carboxymethoxyphenyl)-2-(4-sulfonyl)-2*H*-tetrazolium (MTS; Promega; Madison, WI, USA); phenazine methosulfate (PMS; Sisco Research Laboratories; Andheri, Mumbai, India); cDNA Reverse Transcription kit (Applied Biosystems, Grand Island, NY, USA); TRIzol reagent (Ambion, Austin, TX, USA); ThiolTracker™ Violet, adenosine triphosphatase (ATP) determination kit, and 3,8-phenanthridinediamine,5-(6-triphenylphosphoniumhexyl)-5,6-dihydro-6-phenyl (MitoSOX™ red; Molecular Probes, Carlsbad, CA, USA); fetal bovine serum (FBS, Gibco); SuperSignal™ West Pico PLUS Chemiluminescent Substrate (Thermo Fischer Scientific, Waltham, MA, USA); Optilux microtest plates for ATP determination and Cell-Tak™ (Corning^®^, Tewksbury, MA, USA); cell lysis buffer and antibodies against AMPKα (AMP-activated protein kinase-α; CST, cat. no. 5831); mammalian target of rapamycin (mTOR; CST, cat. no. 2983) or their phosphorylated counterpart P-AMPKα (CST, cat. no. 2535); P-mTOR (CST, cat. no. 5536); peroxisome proliferator-activated receptor-gamma coactivator (PGC1α; ABclonal, cat. no. A12348); glucose transporter 1 (GLUT1; ABclonal, cat. no. A6982); β-actin (CST, cat. no. 4970); horseradish peroxidase (HRP)-linked anti-rabbit secondary IgG antibody (ABclonal, cat. no. AS014) from Abclonal Science, Inc. (Woburn, MA, USA) or Cell Signaling Technology (Danvers, MA, USA). All reagents, instruments, and analyzing software for droplet digital polymerase chain reaction (ddPCR), western blotting, ChemiDoc XRS+ system, Precision Plus Protein™ All Blue Prestained Protein Standards (Bio-Rad, cat. no. 1610373) were from Bio-Rad Laboratories (Hercules, CA, USA), and NanoDrop™ One^C^ was from Thermo Scientific (Waltham, MA, USA). Amphotericin B (Ampho B, 10 mM) and miltefosine (hexadecylphosphocholine [HePC], 10 mM in DMSO) were stored at −20 °C until use.

### Parasite culture

*L. donovani* promastigotes (MHOM/IN/1983/AG83) were maintained for up to ten passages at 24 °C in M199 medium (Phenol Red^+^) with 10% FBS, penicillin G (50 IU/ml), and streptomycin (50 μg/ml); cells were subcultured every 72 h, inoculum being 1 × 10^6^ cells/ml.

### Animal maintenance

Swiss albino mice (20–25 g) of either sex were obtained from the State Centre for Laboratory Animal Breeding (under West Bengal Livestock Development Corporation Ltd.), Kalyani (registration no. 1913/GO/Bt/S/16/CPCSEA), and housed in the animal facility of the Institute of Postgraduate Medical Education and Research (IPGMER), Kolkata, India, under controlled temperature (22 ± 5 °C), humidity (30–70%), and a 12 h light/dark cycle. The animals were provided with standard rodent chow and water *ad libitum*. These experiments were approved by the Institutional Animal Ethics Committee of IPGMER, Kolkata (registration no. 544/PO/c/02/CPCSEA). To obtain murine peritoneal macrophages, mice underwent cervical dislocation and were killed as per recommendations of the Committee for the Purpose of Control and Supervision of Experiments on Animals (CCSEA).

### Evaluation of anti-promastigote activity

The antileishmanial activity of conventional antileishmanials, Ampho B (0–100 nM) and HePC (0–25 µM), were measured in log-phase promastigotes (1 × 10^5^ cells/200 µl/well) for 48 h at 24 °C in M199 medium, using a modified MTS–PMS cell viability assay [12, 13].

### Establishment of infection and assessment of anti-amastigote efficacy

Resident murine peritoneal macrophages were isolated, seeded (~2 × 10^6^/ml/well) and allowed to adhere overnight in 12-well plates at 37 °C, 5% CO_2_ in Roswell Park Memorial Institute Medium (RPMI) (Phenol Red^+^) supplemented with 10% FBS, penicillin G (50 IU/ml), and streptomycin (50 μg/ml). Infection was allowed to be established for 24 h using 5–7-day-old stationary metacyclic promastigotes at a ratio of 10:1 (parasite:macrophage); Ampho B (0–50 nM) or HePC (0–5 µM) were added for an additional 48 h at 37 °C, 5% CO_2_ [14]. Subsequently, RNA (1.0 µg) extracted using the TRIzol method was converted to cDNA using a one-step cDNA reverse transcription kit, and absolute quantification was performed by droplet digital PCR (ddPCR) using amastigote-specific primers for *A2* and quantified in terms of copies/µl [13]. The primers (sourced by NCBI Primer-BLAST and confirmed by UCSC In-Silico PCR) included amastigote-specific genes *A2* (F, 5′-CTGCAGGCTGTTGACGTTTC-3′; R, 5′-AAGGTTTGCCTCGTCACCAT-3′).

### Evaluation of safety index

The safety index was evaluated in murine peritoneal macrophages isolated from Swiss albino mice (5 × 10^5^ cells/200 µl/well) and J774A.1 cell line (2 × 10^4^ cells/200 µl/well) maintained in RPMI medium and supplemented with 10% FBS, penicillin G (50 IU/ml), and streptomycin (50 μg/ml). Following incubation with Ampho B (0–10 µM) or HePC (0–250 µM) for 48 h at 37 °C, 5% CO_2_, the selectivity/safety index (SI) was quantified by the MTS–PMS assay [12, 14].

### Effect of conventional antileishmanials upon the redox status of *L. donovani* promastigotes

#### Measurement of generation of reactive oxygen species (ROS)

The effect of Ampho B (0–100 nM, 1 h or 3 h, 37 °C) or HePC (0–10 µM, 1 h or 3 h, 37 °C) on generation of ROS was measured in log-phase promastigotes (1 × 10^6^ cells/ml, 0.5 ml, using 2,7-dichlorodihydrofluoresceindiacetate (H_2_DCFDA; 100 µM, 45 min, 37 °C). Fluorescence was acquired on a flow cytometer (FACS Verse, BD Biosciences, San Jose, CA, USA), with hydrogen peroxide (H_2_O_2_ 1 mM, 1 h, 37 °C) being the positive control [13].

#### Measurement of nonprotein thiols

Levels of nonprotein thiols were determined by flow cytometry in Ampho B-treated (0–100 nM, 1 h or 3 h, 37 °C) or HePC-treated (0–10 µM, 1 h or 3 h, 37 °C) log-phase promastigotes (AG83, 1 × 10^6^ cells/ml, 0.5 ml) using ThiolTracker™ Violet (TV; 2.5 µM, 30 min, 37 °C), keeping *N*-ethylmaleimide (NEM; 100 µM, 30 min, 37 °C) as the positive control [13].

### Effect of antileishmanials on mitochondrial functions in *L. donovani* promastigotes

#### Measurement of mitochondrial generation of superoxide

Log-phase promastigotes (1 × 10^6^ cells/ml, 0.5 ml) prestained with MitoSOX™ (2.5 µM, 1 h, 37 °C), were incubated with Ampho B (0–100 nM), or HePC (0–10 µM) for 1 h or 3 h at 37 °C, and fluorescence was acquired in a flow cytometer. Oligomycin (Oligo; 1 µM, 1 h, 37 °C) served as the positive control [13].

#### Measurement of levels of ATP

Levels of ATP were measured in log-phase promastigotes (1 × 10^6^ cells/ml) treated with Ampho B (0–100 nM, 1 h or 3 h, 37 °C) or HePC (0–10 µM, 1 h or 3 h, 37 °C) and measured by a chemiluminescence method utilizing an ATP determination kit as per the manufacturer’s instructions (Molecular Probes, Carlsbad, CA, USA), using a flurimeter (Spectramax M2e, Molecular Devices, San Jose, CA, USA); Oligomycin (Oligo; 10 µM, 1 h, 37 °C) served as the positive control [13].

#### Isolation of mitochondria and complex assays

Mitochondria from log-phase AG83 promastigotes (1–2 × 10^9^) were isolated following hypotonic lysis and differential centrifugation, after which they were stored at −20 °C until use [15].

#### Complexes I–III (NADH cytochrome c reductase) coupled assay

In mitochondria isolated from promastigotes, the activity of complexes I–III (NADH cytochrome c reductase) was measured following the addition of Ampho B (0–100 nM) or HePC (0–10 µM) by the NADH-supported reduction of ferricytochrome c to ferrocytochrome c, increasing absorbances being measured at 550 nm (Spectramax M2e). The enzyme activity was calculated in terms of nmol of Cyt c reduced/min/mg of protein, with rotenone (0.25 mM) being the positive control [15].

#### Complexes II–III (succinate Cyt c reductase) coupled assay

The activity of complexes II–III (succinate Cyt c reductase) was measured in isolated mitochondria following treatment with Ampho B (0–100 nM) or HePC (0–10 µM) by the succinate-supported reduction of ferricytochrome c to ferrocytochrome c, increasing absorbances being measured at 550 nm using Spectramax M2e; thenoyltrifluoroacetone (TTFA; 0.4 mM) was the positive control [15].

### Assessment of metabolic bioenergetics in *L. donovani* promastigotes treated with conventional antileishmanials

Real-time measurements of mitochondrial respiration and glycolytic activity of log-phase promastigotes (2 × 10^6^/180 µl/well) following treatment with Ampho B (0–100 nM, 1 h, 37 °C) or HePC (0–10 μM, 1 h, 37 °C) were performed using Seahorse Metabolic Analyzer XFp (Agilent Technologies, Santa Clara, CA, USA), in terms of their oxygen consumption rate (OCR; pmol/min/2 × 10^6^ parasites) and extracellular acidification rate (ECAR; mpH/min/2 × 10^6^ parasites) respectively, as per the manufacturer’s instructions [13, 16].

To identify the major substrate(s) utilized by promastigote mitochondria as their fuel sources for the tricarboxylic acid (TCA) cycle, the substrate oxidation stress test was performed. Inhibitors used were BPTES 200 µM (inhibition of glutamine through glutaminase 1), etomoxir 200 µM (for long-chain fatty acids (LCFAs) through inhibition of carnitine palmitoyl transferase 1a (CPT1a), and UK5099 200 µM (for pyruvate, through inhibition of the mitochondrial pyruvate carrier [MPC]) [17, 18]. Data were analyzed using Seahorse Wave software, version 2.6.1, along with the XFp Mito stress test report generator (Agilent Technologies).

### mRNA expression of major bioenergetic regulatory genes in *L. donovani*-infected macrophages

Absolute quantification was performed by ddPCR as per manufacturer’s instructions (Bio-Rad Laboratories, Hercules, CA, USA) using primers (Table 1, sourced from NCBI Primer-BLAST and confirmed by UCSC In-Silico PCR). They included genes pertaining to (i) mitochondrial oxidative phosphorylation, (ii) rate-limiting enzymes of glycolysis, and (iii) regulators of major metabolic bioenergetics [9].

**Table 1:** Primers and amplification conditions of major bioenergetic regulatory genes for ddPCR

Serial no.	Target genes	Forward sequences (5′–3′)	Reverse sequences (5′–3′)	Annealing temperature (°C)
1	*Cox IV* (cytochrome c oxidase)	TGGCAAGAGAGCCATTTCTACT	GTGGGGAAAGCATAGTCTTCAC	67
2	*Atp synthase* (subunit b)	GTGGTTCGTTCATCTGTCCATA	GTATTTGCTGGTGTTGGTGAGA	62
3	*Pdk1* (Pyruvate dehydrogenase kinase 1)	GGACTTCGGGTCAGTGAATGC	TCCTGAGAAGATTGTCGGGGA	66
4	*Ldh* (Lactate dehydrogenase)	TGTCTCCAGCAAAGACTACTGT	GACTGTACTTGACAATGTTGGGA	62
5	*HkII* (Hexokinase II)	TGATCGCCTGCTTATTCACGG	AACCGCCTAGAAATCTCCAGA	62
6	*Pfk* (Phosphofructo kinase)	GGAGGCGAGAACATCAAGCC	CGGCCTTCCCTCGTAGTGA	62
7	*Pkm2* (Pyruvate Kinase M2)	TTGCAGCTATTCGAGGAACTCCG	CACGATAATGGCCCCACTGC	67
8	*Glut1*(glucose transporter 1)	CCAGCTGGGAATCGTCGTT	AAGTCTGCATTGCCCATGAT	58
9	*Ampk* (AMP-dependent protein kinase)	CAGACTTTTGGGGACTTTGCC	ATGATGTGAGGGTGCCTGAAC	62
10	*Lkb1* (Liver kinase B1)	GGCATGGACACCTTCATCCA	GAGTCCAGCACCTCCTTCAC	60
11	*Sirt1* (Sirtuin 1)	AGAACCACCAAAGCGGAAA	TCCCACAGGAGACAGAAACC	62
12	*Pgc1α* (Peroxisome proliferator-activated receptor gamma coactivator 1α)	AGCCGTGACCACTGACAACGAG	GCTGCATGGTTCTGAGTGCTAAG	67
13	*Mtor* (mammalian target of rapamycin)	CGGGACTACAGAGAGAAGAAGA	CATCAACGTCAGGTGGTGGTCATAG	62

All the primers were sourced from NCBI Primer-BLAST (https://www.ncbi.nlm.nih.gov/tools/primer-blast); specificity was confirmed by UCSC In-Silico PCR) and included major metabolic bioenergetic regulatory genes or rate-limiting enzymes of mitochondrial oxidative phosphorylation or glycolysis [9]. The annealing temperatures were optimized on the basis of the best separation between positive and negative droplets of uninfected and infected samples in ddPCR

Briefly, cDNA (50 ng) was added to ddPCR EvaGreen Supermix (Bio-Rad Laboratories), containing 100 nM of each forward and reverse primer in a final volume of 20 μl (following standardization of annealing temperature). This was followed by generation of droplets using a QX200 droplet generator. Subsequently, PCR was performed (40 cycles at 95 °C for 5 min, 95 °C for 30 s, annealing [as listed in Table 1] for 1 min, followed by a final extension at 90 °C for 5 min). The resultant products were then scanned on a QX200 Droplet Reader, and the data were analyzed using QuantaSoft software (version 1.7.4) and quantified in terms of copies/µl [13].

### Immunoblotting

Protein lysates prepared from *L. donovani*-infected peritoneal macrophages were incubated with Ampho B (0–50 nM) or HePC (0–5 µM) for 48 h at 37 °C, 5% CO_2_. Briefly, cells were removed by gentle scraping and resuspended in ice-cold lysis buffer, followed by sonication and centrifugation (18,894*g*, 30 min, 4 °C). The resultant supernatants were collected and total protein was estimated by Bradford assay [14]. After boiling (100 °C, 5 min), the supernatant in sodium dodecyl sulfate (SDS) sample buffer along with equal amounts of proteins (40 µg/well) were resolved on SDS–polyacrylamide mini gels (SDS–PAGE 5–10%) and transferred to nitrocellulose membranes. Following blocking of the nonspecific binding sites by incubating for 2 h with Tris-buffered saline containing 5% bovine serum albumin (TBS–BSA), they were incubated overnight at 4 °C with antibodies against GLUT1, PGC1α, AMPKα, mTOR, or their phosphorylated counterparts, P-AMPKα and P-mTOR. All primary antibodies were raised in rabbit (1:500 dilution in 3% TBS–BSA, except β-actin, 1:1000 dilution in 3% TBS–BSA). Following washes with TBS–Tween (0.1%), binding was detected using horseradish peroxidase (HRP)-linked anti-rabbit secondary IgG (1:2000 dilution in 3% TBS–BSA 2 h, room temperature). The immunoreactive bands were detected using West Pico Plus chemiluminescent substrate, and images were captured using BioRad ChemiDoc™ XRS+ , followed by densitometric analysis using Image Lab™ software, version 3.0.

Each blot was initially probed with the phosphorylated antibody, followed by stripping, by incubating in a stripping buffer (0.1 M glycine, pH 2.5, 37 °C, 60 min), and then reprobed with its counterpart (total). Finally, after another round of stripping, the membranes were reprobed with β-actin as the loading control. Densitometric analysis was performed, and phosphorylated proteins were normalized with respect to their respective total protein, while PGC1α and GLUT1 were normalized with β-actin.

### Statistical analysis

Each experiment was performed at least thrice in duplicates; results were expressed as mean (± standard error [SE]). Statistical analysis was evaluated by Kruskal Wallis multiple comparison test (one-way analysis of variance [ANOVA]) or Bonferroni multiple comparison test (two-way ANOVA) for nonparametric data using GraphPad Prism, version 8.4.2 (GraphPad Software Inc., La Jolla, CA, USA); *P* < 0.05 was considered as statistically significant.

## Results

### Leishmanicidal efficacy of conventional antileishmanials (Ampho B and HePC)

The anti-promastigote efficacy of conventional antileishmanials, Ampho B and HePC, was initially checked in AG83 promastigotes, and their 50% inhibitory concentration (IC_50_) was derived, being 52.30 ± 04.05 nM and 5.80 ± 0.53 µM, respectively, while their 90% inhibitory concentration (IC_90_) was 93.32 ± 1.68 nM and 11.66 ± 0.66 µM, respectively (Supplementary Fig. 1A(i), B(i)). Accordingly, experiments were performed in promastigotes using the IC_50_–IC_90_ range of Ampho B (12.5–100 nM) and HePC (1.25–10 µM).

The anti-amastigote activity was confirmed by quantification of parasite load using ddPCR employing the amastigote-specific *A2*. The IC_50_ of Ampho B and HePC was 9.42 ± 0.38 nM and 0.89 ± 0.38 µM, respectively, whereas their IC_90_ was 50.13 ± 0.76 nM and 4.73 ± 0.51 µM, respectively (Supplementary Fig. 1A(ii), B(ii)). Accordingly, all amastigote-based experiments were performed in the IC_50_–IC_90_ range of Ampho B (10–50 nM) and HePC (1–5 µM). The 50% cytotoxic concentration (CC_50_) of Ampho B in J774.A1 and murine peritoneal macrophages was > 10 µM, whereas for HePC, it was 125.9 ± 16.18 μM and 151.7 ± 08.13 μM, respectively (Supplementary Fig. 1A(iii), B(iii); Supplementary Table 1).

### Ampho B and HePC caused a redox imbalance in promastigotes

**Fig. 1:**
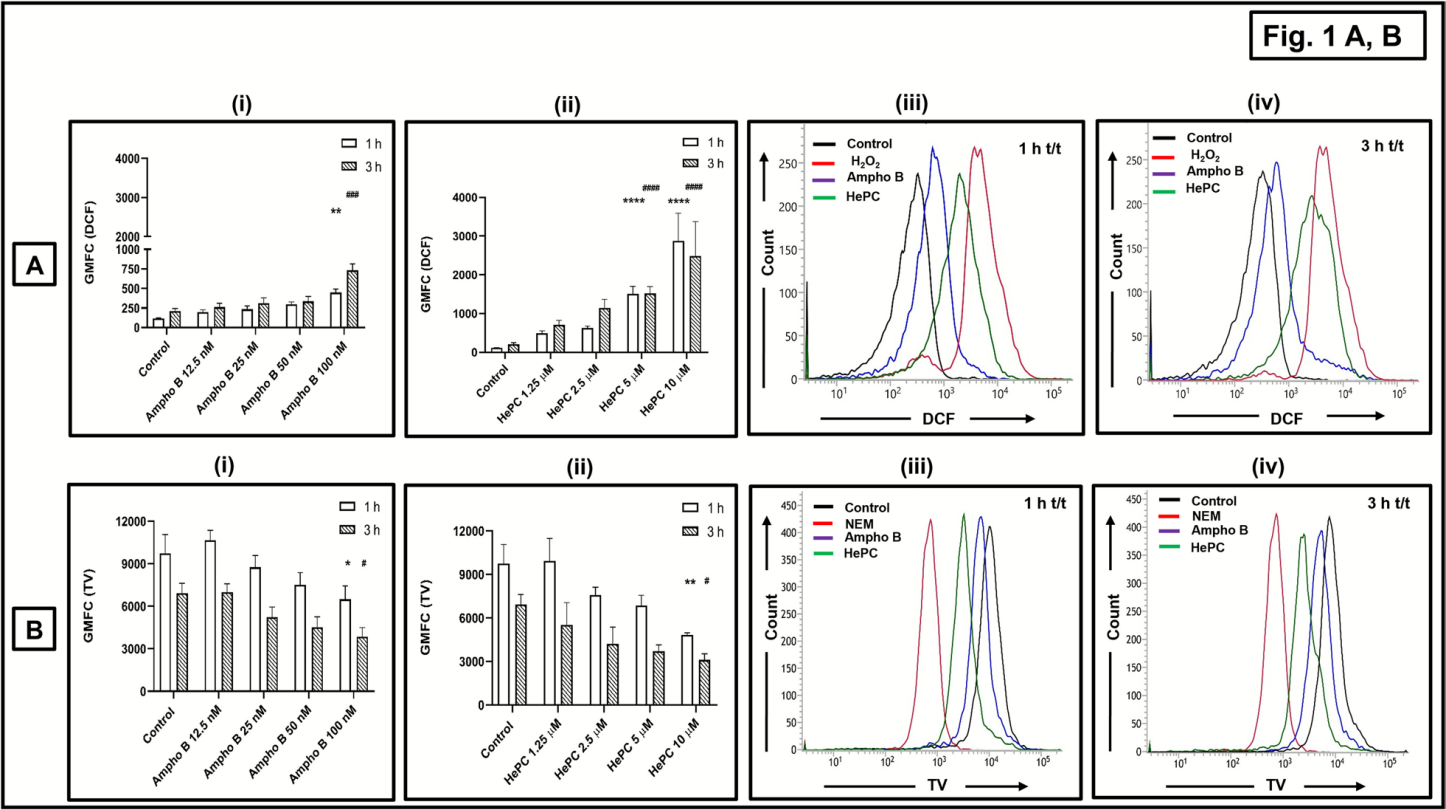
Effect of conventional antileishmanials on cellular redox status of *Leishmania donovani* promastigotes. **A**, **B**—**i**, **ii** Log-phase promastigotes (AG83, 5 × 10^5^ cells/0.5 ml) were incubated with amphotericin B (Ampho B 0–100 nM) **(i)** or miltefosine (HePC 0–10 µM) **(ii)** and then labeled with H_2_DCFDA (100 µM, 45 min, 37 °C) (**A**) and ThiolTracker Violet (TV; 2.5 µM, 30 min, 37 °C) (**B**). Fluorescence was acquired and analyzed as described in the “Methods” section. Data are expressed as the mean (± standard error [SE]) of the geometrical mean fluorescence channel (GMFC) of at least three experiments in duplicates; ^*^P < 0.05, ^**^P < 0.01, ^***^P < 0.001, and ^****^P < 0.0001 as compared with baseline at 1 h and ^#^*P* < 0.05, ^##^*P* < 0.01, ^###^*P* < 0.001, and ^####^*P* < 0.0001 as compared with baseline at 3 h. **A**, **B**—**iii, iv** Representative histogram profiles of AG83 promastigotes (baseline) incubated with Ampho B 100 nM or HePC 10 µM for 1 h (**iii**) or 3 h (**iv**) or H_2_O_2_ (1 mM, 1 h, 37 °C) (**A**) or NEM (100 μM, 30 min, 37 °C) (**B**) and stained with H_2_DCFDA (**A**) or ThiolTracker™ Violet (TV) (**B**) as described in the “Methods” section

Ampho B (12.5–100 nM) caused a dose-dependent increase in DCF fluorescence that increased significantly only at 100 nM at 1 h (3.79-fold, *P* < 0.01) and 3 h (3.52-fold, *P* < 0.001; Fig. 1A(i); Table 2). Similarly, HePC (1.25–10.0 µM) caused a dose-dependent enhancement in the generation of ROS that increased significantly with 5.0 μM at 1 h (12.75-fold, *P* < 0.0001) and 3 h (7.34-fold, *P* < 0.0001). Similarly, with 10.0 µM HePC, there was a significant increase at 1 h (24.29-fold, *P* < 0.0001) and 3 h (11.96-fold, *P* < 0.0001; Fig. 1A(ii); Table 2). Taken together, irrespective of the time points, the generation of ROS by HePC was consistently higher with Ampho B (Fig. 1A). H_2_O_2_ (1 mM, 1 h) served as positive control, which caused a 39.22-fold (*P* < 0.0001) enhanced generation of ROS (Table 2); with a 3 h incubation, there was a gate shift, suggesting toxicity.

**Table 2:** Effect of conventional antileishmanials on cellular redox status of *L. donovani* promastigotes

Groups	Generation of ROS (GMFC DCF)		Non-protein thiols (GMFC TV)	
	1 h	3 h	1 h	3 h
Control	118.12 ± 4.72	207.25 ± 36.66	9747.33 ± 1314.99	6924.5 ± 701.35
Positive control	4632.75 ± 196.25^****^ (H_2_O_2_ 1 mM)	NA	1484 ± 238.89^****^ (NEM 100 µM)	NA
Ampho B, 12.5 nM	196.80 ± 30.09	259.60 ± 50.91	10663 ± 699	6993.66 ± 587.60
Ampho B, 25 nM	234.2 ± 39.82	310.20 ± 68.59	8759.5 ± 822.11	5239 ± 702.19
Ampho B, 50 nM	298.83 ± 28.67	334.80 ± 62.12	7519.5 ± 853.30	4520 ± 736.31
Ampho B, 100 nM	448.60 ± 45.05^**^	731.40 ± 81.29^###^	6498.50 ± 943.77^*^	3853.25 ± 639.88^#^
HePC, 1.25 µM	495.80 ± 55.35	704.25 ± 117.14	9929.5 ± 1546.51	5520 ± 1532.76
HePC, 2.5 µM	628.75 ± 46.20	1149.50 ± 219.35	7574 ± 554.22	4217.66 ± 1145.08
HePC, 5.0 µM	1507 ± 195.21^****^	1522.50 ± 171.79^####^	6850 ± 712.13	3704.33 ± 443.85
HePC, 10.0 µM	2870 ± 722.84^****^	2480.60 ± 895.14^####^	4832.5 ± 152.31^**^	3112.66 ± 421.36^#^

Log-phase promastigotes (AG83, 5 × 10^5^ cells/0.5 ml) were incubated with Ampho B (0–100 nM, 1 h or 3 h, 37 °C) or HePC (0–10 µM, 1 h or 3 h, 37 °C) or H_2_O_2_ (1 mM, 1 h, 37 °C) or NEM (100 µM, 30 min, 37 °C) and then labeled with H_2_DCFDA (100 µM, 45 min, 37 °C) or ThiolTracker™ Violet (TV 2.5 µM, 30 min, 37 °C). Fluorescence was acquired and analyzed as described in the “Methods” section. Data are expressed as the mean (± standard error [SE]) of GMFC of at least three different experiments in duplicates; ^*^P < 0.05, ^**^P < 0.01, ^***^P < 0.001, and ^****^P < 0.0001 as compared with baseline at 1 h and ^#^*P* < 0.05, ^##^*P* < 0.01, ^###^*P* < 0.001, and ^####^*P* < 0.0001 as compared with baseline at 3 h

Ampho B (12.5–100 nM) significantly depleted the nonprotein thiols at its highest concentration (100 nM) at 1 h (1.49-fold, *P* < 0.05) and 3 h (1.79-fold, *P* < 0.05) (Fig. 1B(i); Table 2). Similarly, HePC at its highest concentration (10 µM) depleted thiols at 1 h (2.01-fold, *P* < 0.01) and 3 h (2.22-fold, *P* < 0.05 (Fig. 1B(ii); Table 2). The proportion of depletion of nonprotein thiols was significantly higher with HePC than with  Ampho B (Fig. 1B); NEM (100 µM, 30 min) served as the positive control and caused a 6.56-fold (*P* < 0.0001) decrease in nonprotein thiols (Table 2).

### Impairment of mitochondrial functions by Ampho B and HePC

**Fig. 2:**
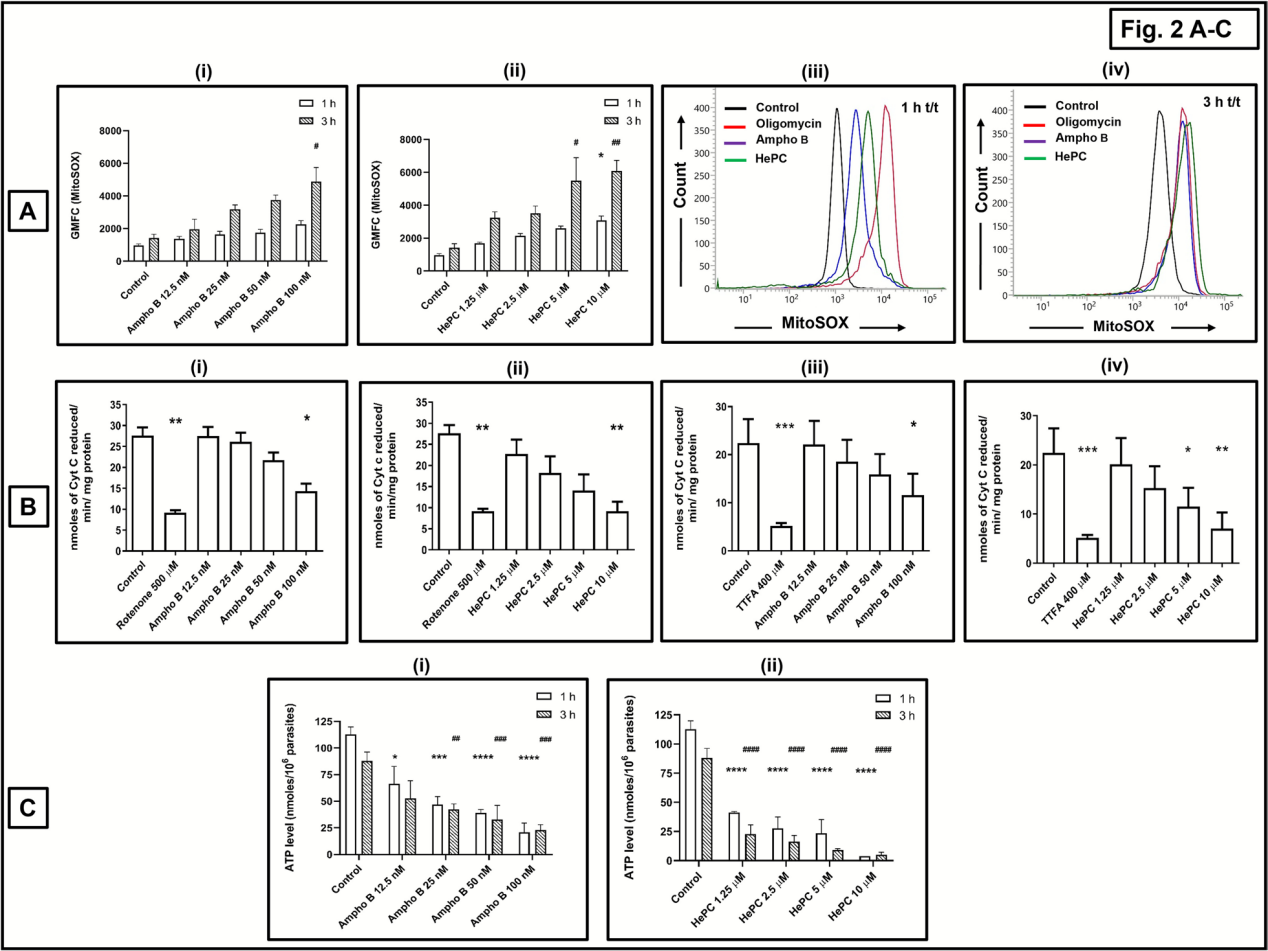
Effect of conventional antileishmanials upon mitochondrial functions in *Leishmania donovani* promastigotes. **A**—**i**, **ii** Log-phase promastigotes (AG83, 5 × 10^5^ cells/0.5 ml) were incubated with amphotericin B (Ampho B 0–100 nM) (**i**) or miltefosine (HePC 0–10 µM) (**ii**) and then labeled with MitoSOX™ (2.5 µM, 1 h, 37 °C). Fluorescence was acquired and analyzed as described in the “Methods” section. Data are expressed as the mean (± standard error [SE]) of GMFC of at least three experiments in duplicates; ^*^P < 0.05 as compared with baseline at 1 h and ^#^*P* < 0.05 and ^##^*P* < 0.01 as compared with baseline at 3 h. **A**—**iii**, **iv** Representative histogram profiles of AG83 promastigotes (baseline) treated with Ampho B 100 nM or HePC 10 µM for 1 h (**iii**) or 3 h (**iv**) or oligomycin (Oligo; 1 µM, 1 h, 37 °C) and stained with MitoSOX™ as described in the “Methods” section. **B** Effect of Ampho B (0–100 nM;** i**, **iii**) or HePC (0–10 µM; **ii**, **iv**) or rotenone 500 µM (**i**, **ii**) or TTFA 400 µM (**iii**, **iv**) on enzymatic activities of mitochondrial respiratory chain, namely complexes I–III (NADH cytochrome c reductase) (**i**, **ii**) and complexes II–III (succinate Cyt c reductase) (**iii**, **iv**) activities were assessed in the crude mitochondria sourced from *Leishmania donovani* (AG83) promastigotes as described in the “Methods” section. Data are expressed as mean (± standard error [SE]) of nmol of Cyt c reduced/min/mg protein of at least three experiments in duplicate; ^*^P < 0.05, ^**^P < 0.01, and ^***^P < 0.001 as compared with control. **C**—**i**, **ii** Levels of ATP were determined in AG83 promastigotes (1 × 10^6^) following treatment with Ampho B (0–100 nM, 1 h or 3 h, 37 °C) or HePC (0–10 µM, 1 h or 3 h, 37 °C) or oligomycin (Oligo; 1 µM, 1 h, 37 °C). The ATP content was determined by the luciferin/luciferase reaction as described in the “Methods” section. The results are expressed as the mean (± standard error [SE]) of nmol/10^6^ promastigotes of at least three experiments in duplicates; ^*^P < 0.05, ^**^P < 0.01, ^***^P < 0.001, and ^****^P < 0.0001 as compared with baseline at 1 h and ^#^*P* < 0.05, ^##^*P* < 0.01, ^###^*P* < 0.001, and ^####^*P* < 0.0001 as compared with baseline at 3 h

Mitochondria is one of the major sources of cellular ROS, with superoxide (O_2_^•−^) being the prime contributor; accordingly, inhibition of the mitochondrial electron transport chain (ETC) leads to an enhanced generation of superoxide [19]. Ampho B (12.5–100 nM) dose dependently enhanced the generation of superoxide, maximally evident at 100 nM, 3 h (3.43-fold, *P* < 0.5) (Fig. 2A(i); Table 3). Similarly, HePC (1.25–10 µM) caused a gradual increase in the generation of superoxide (Fig. 2A(ii); Table 3), being significant at 10 µM at 1 h (3.17-fold, *P* < 0.05) and 3 h (4.28-fold, *P* < 0.01). In sum, both Ampho B and HePC substantially enhanced the generation of superoxide, with HePC producing a significantly higher proportion than Ampho B (Fig. 2A). Oligomycin (Oligo; 1 µM, 1 h) served as the positive control, and caused a 5.21-fold (*P* < 0.01) increase (Table 3).

**Table 3:** Evaluation of mitochondrial functions in *L. donovani* promastigotes treated with conventional antileishmanials

Groups	Mitochondrial superoxide (GMFC MitoSOX)		Levels of ATP (nmol/10^6^ parasites)	
	1 h	3 h	1 h	3 h
Control	968.66 ± 92.74	1421.66 ± 233.27	112.74 ± 7.06	88.05 ± 8.16
Positive control	5053.91 ± 1076.15^**^ (Oligomycin 1 µM)	NA	13.70 ± 3.43^****^ (Oligomycin 10 µM)	NA
Ampho B, 12.5 nM	1377.25 ± 137.08	1947.5 ± 627.86	66.52 ± 16.18^*^	52.74 ± 16.60
Ampho B, 25 nM	1636.25 ± 193.43	3192.5 ± 255.47	46.92 ± 7.35^***^	42.45 ± 5.06^##^
Ampho B, 50 nM	1747.25 ± 196.09	3753.5 ± 294.16	39.26 ± 2.97^****^	32.83 ± 13.23^###^
Ampho B, 100 nM	2272 ± 213.56	4887 ± 858.52^#^	20.94 ± 8.50^****^	23.03 ± 4.88^###^
HePC, 1.25 µM	1697.4 ± 49.99	3242 ± 361.42	41.39 ± 0.84^****^	22.76 ± 7.85^####^
HePC, 2.5 µM	2141.5 ± 141.52	3510.5 ± 443.11	27.66 ± 9.89^****^	16.29 ± 5.23^####^
HePC, 5.0 µM	2607 ± 121.20	5493.33 ± 1395.24^#^	23.47 ± 11.68^****^	9.11 ± 1.29^####^
HePC, 10.0 µM	3070.75 ± 272.34^*^	6089.66 ± 639.99^##^	3.77 ± 0.15^****^	5.17 ± 2.08^####^

Log-phase promastigotes (AG83, 5 × 10^5^ cells/0.5 ml) were incubated with Ampho B (0–100 nM, 1 h or 3 h, 37 °C) or HePC (0–10 µM, 1 h or 3 h, 37 °C) or oligomycin (Oligo; 1 µM, 1 h, 37 °C) and then labeled with MitoSOX™ (2.5 µM, 1 h, 37 °C). Fluorescence was acquired and analyzed as described in the “Methods” section. Data are expressed as the mean (± standard error [SE]) of GMFC of at least three different experiments in duplicates; ^*^P < 0.05 and ^**^P < 0.01 as compared with baseline at 1 h and ^#^*P* < 0.05, ^##^*P* < 0.01 as compared with baseline at 3 h

Levels of ATP were determined in AG83 promastigotes (1 × 10^6^) following treatment with Ampho B (0–100 nM, 1 h or 3 h, 37 °C) or HePC (0–10 µM, 1 h or 3 h, 37 °C) or oligomycin (Oligo; 10 µM, 1 h, 37 °C). The ATP content was determined by the luciferin/luciferase reaction as described in the “Methods” section. The results are expressed as the mean (± standard error [SE]) of nmol/10^6^ promastigotes of at least three different experiments in duplicates; ^*^P < 0.05, ^**^P < 0.01, ^***^P < 0.001, and ^****^P < 0.0001 as compared with baseline at 1 h and ^#^*P* < 0.05, ^##^*P* < 0.01, ^###^*P* < 0.001, and ^####^*P* < 0.0001 as compared with baseline at 3 h

To explore whether the enhanced generation of superoxide was secondary to inhibition of the mitochondrial ETC, the activities of NADH cytochrome c reductase (complex I–III) and succinate cytochrome c reductase (complex II–III), were examined in crude mitochondrial isolates sourced from AG83 promastigotes. Ampho B at IC_90_ (100 nM) inhibited the complex I–III activity by 1.93-fold (*P* < 0.05 (Fig. 2B(i); Table 4), whereas HePC (5.0 and 10.0 µM) caused a 1.96- and 3.01-fold decrease in complex I–III activity, respectively (Fig. 2B(ii); Table 4). Likewise, Ampho B at the IC_90_ (100 nM) inhibited complex II–III activity by 1.94-fold (*P* < 0.05 (Fig. 2B(iii); Table 4), whereas HePC (5.0 and 10.0 µM) caused a 1.95- (*P* < 0.05) and 3.19-fold (*P* < 0.01) decrease in activity of complex II–III, respectively (Fig. 2B(iv); Table 4). The positive controls for complexes I–III and II–III, rotenone (500 µM) and TTFA (400 µM), respectively, demonstrated significant inhibition (Table 4). Overall, inhibition of the mitochondrial ETC by HePC was significantly greater than by Ampho B.

**Table 4:** Impact of conventional antileishmanials upon the enzymatic activities of mitochondrial electron transport chain (ETC) sourced from *L. donovani* promastigotes

Groups	Complex I–III (NADH cytochrome c reductase) assay (nmol of Cyt C reduced/min/mg protein)	Complex II–III (succinate cytochrome c reductase) assay (nmol of Cyt C reduced/min/mg protein)
Control	27.60 ± 01.95	22.45 ± 04.95
Positive control	9.16 ± 0.58^**^ (rotenone 500 µM)	5.16 ± 0.58^***^ (TTFA 400 µM)
Ampho B, 12.5 nM	27.52 ± 02.12	22.14 ± 04.88
Ampho B, 25 nM	26.12 ± 02.12	18.54 ± 04.55
Ampho B, 50 nM	21.73 ± 01.85	15.91 ± 04.21
Ampho B, 100 nM	14.28 ± 01.84^*^	11.55 ± 04.49^*^
HePC, 1.25 µM	22.74 ± 03.39	20.11 ± 05.34
HePC, 2.5 µM	18.22 ± 03.94	15.25 ± 04.47
HePC, 5.0 µM	14.08 ± 03.80	11.49 ± 03.81^*^
HePC, 10.0 µM	09.15 ± 02.20^**^	07.02 ± 03.32^**^

The effects of Ampho B (0–100 nM) or HePC (0–10 µM) upon the activities of complexes I–III and II–III were evaluated as described in the “Methods” section. Data are expressed as mean (± standard error [SE]) of nmol of Cyt c reduced/min/mg protein of at least three different experiments in duplicates; ^*^P < 0.05, ^**^P < 0.01, and ^***^P < 0.001 as compared with baseline

As oxidative stress and mitochondrial/cellular dysfunction influence the energy demand, the effects of Ampho B and HePC upon ATP levels were evaluated. Ampho B (12.5–100 nM) dose dependently depleted ATP at 1 h and 3 h (Fig. 2C(i); Table 3), as did HePC (Fig. 2C(ii); Table 3). The degree of depletion by HePC was significantly greater than by Ampho B. The assay specificity was confirmed using oligomycin (10 μM, 1 h), an established inhibitor of the F_0_F_1_-ATPase complex, which significantly depleted ATP by 8.23-fold (*P* < 0.0001) (Table 3).

### Effect of Ampho B and HePC upon mitochondrial bioenergetics

Pathogens and mammalian cells meet their energy demand by utilizing two major energy-producing cellular pathways, namely oxidative phosphorylation in mitochondria and glycolysis [20]. Additionally, metabolic bioenergetics is known to influence macrophage polarization, an essential feature for establishment of *Leishmania* infection [8, 11, 21]. It can be envisaged that selective targeting of these major energy-producing pathways could be a potential chemotherapeutic target. Accordingly, the impact of Ampho B and HePC upon mitochondrial oxygen consumption rate (OCR) and glycolytic functions (ECAR) was evaluated in *L. donovani* promastigotes and mammalian macrophages.

**Fig. 3:**
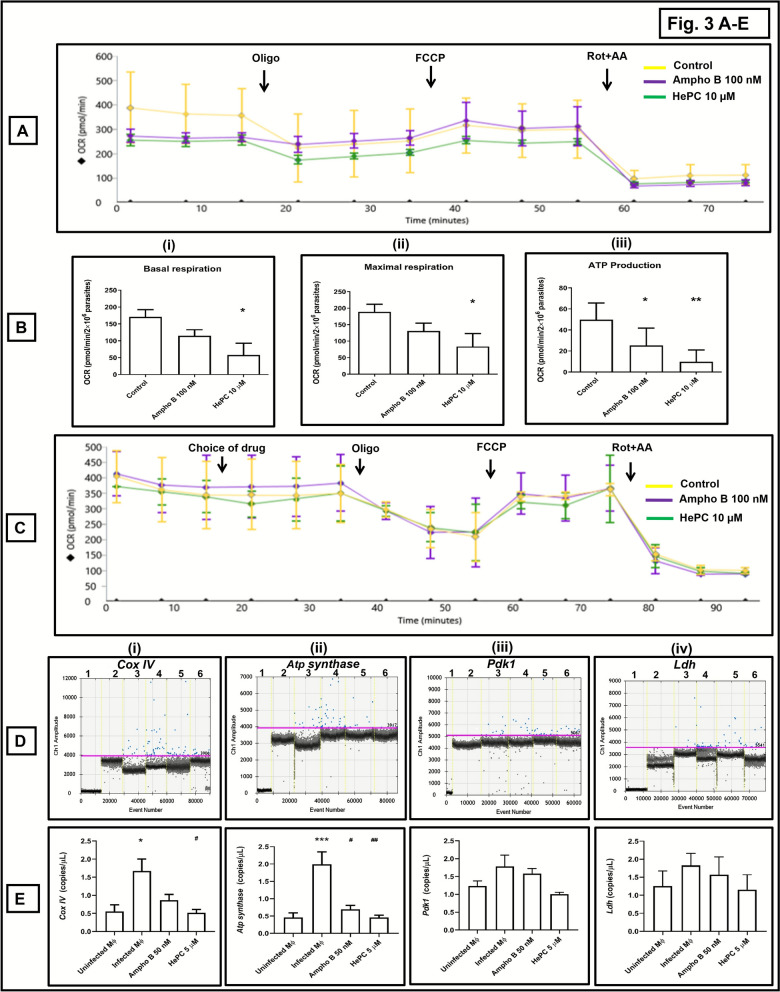
Assessment of mitochondrial bioenergetics in *Leishmania donovani* treated with conventional antileishmanials. **A** Representative profile of mitochondrial respiration (OCR) of at least three independent experiments of log-phase promastigotes (AG83, 2 × 10^6^ cells/well) treated with Ampho B (100 nM, 1 h, 37 °C) or HePC (10 µM, 1 h, 37 °C), following addition of oligomycin (Oligo; 10 µM), FCCP (2 µM), and rotenone–antimycin A (Rot + AA;1 µM, each), as measured by Seahorse Extracellular Flux Analyzer (XFp). **B** Bar graphs of (**i**) basal respiration, (**ii**) maximal respiration, and (**iii**) ATP production. Data are expressed as the mean (± standard error [SE]) of OCR (mpH/min/2 × 10^6^ parasites) of at least three experiments in duplicates; ^*^P < 0.05 and ^**^P < 0.01 as compared with untreated parasites. **C** Representative profile of mitochondrial respiration (OCR) of at least three independent experiments of murine peritoneal macrophages (1 × 10^6^ cells/well) treated with Ampho B (100 nM) or HePC (10 µM) in port A (acute response) followed by addition of oligomycin (Oligo; 10 µM), FCCP (2 µM), and Rot + AA (1 µM, each), as measured by Seahorse Extracellular Flux Analyzer (XFp). **D** Representative profiles of mitochondrial oxidative phosphorylation regulatory genes, namely (**i**) *Cox IV*, (**ii**) *Atp synthase*, (**iii**) *Pdk1*, and (**iv**) *Ldh*, as quantified by ddPCR. One-dimensional plot of droplets measured for fluorescence signals (amplitude indicated on *y*-axis) emitted from respective target-specific primers; lane 1: negative control (NC; nuclease-free water), lane 2: nontemplate controls (NTC), lane 3: uninfected peritoneal macrophages, lane 4: infected peritoneal macrophages treated with Ampho B (lane 5) or HePC (lane 6). EvaGreen-bound positive droplets are shown in blue, while negative droplets are shown in black, with expression of genes quantified as copies/µl as described in the “Methods” section. **E** Bar graphs showing copies/μl of (**i**) *Cox IV*, (**ii**) *Atp synthase* , (**iii**) *Pdk1*, and (**i**) *Ldh* in uninfected macrophages and infected peritoneal macrophages treated with Ampho B or HePC. Data are expressed as the mean (± standard error [SE]) of at least three experiments in duplicates; ^*^P < 0.05, ^**^P < 0.01, and ^***^P < 0.001 as compared with uninfected macrophages; ^#^*P* < 0.05 and ^##^*P* < 0.01 as compared with infected macrophages

In *L. donovani* promastigotes, Ampho B (100 nM, 1 h) caused a depletion in basal respiration and maximal respiration by 32.27% and 30.68%, respectively, which, with HePC (10 µM, 1 h), the percentage of depletion was even higher, being 66.37% (*P* < 0.05) and 55.58% (*P* < 0.05), respectively (Fig. 3A, B(i–ii); Table 5). In corroboration with the chemiluminescence data, Ampho B (100 nM, 1 h) as compared with baseline decreased ATP production by 49.40% (*P* < 0.05) vis-à-vis 80.15% by HePC (10 µM, 1 h, *P* < 0.01 (Fig. 3A, B(i–ii); Table 5). To evaluate the acute response of these two antileishmanials, the drugs were directly added in port A of XFp cartridges (Supplementary Fig. 2A, B). It was consistently observed that inhibition of mitochondrial respiration by HePC was significantly greater than by Ampho B (Supplementary Fig. 2B(iv); Supplementary Table 2A). In murine peritoneal macrophages, Ampho B (100 nM) and HePC (10 µM) did not alter mitochondrial respiration (in terms of basal respiration, maximal respiration, spare respiratory capacity [SRC], ATP-linked respiration, or coupling efficiency (Fig. 3C; Table 6), thus substantiating their nontoxic effect on host macrophages.

**Table 5:** Effect of conventional antileishmanials on mitochondrial oxygen consumption rate (OCR) in *L. donovani* promastigotes

Parameters of mitochondrial respiration (pmol/min/2 × 10^6^ parasites)	Control	Ampho B 100 nM (IC_90_, 1 h)	HePC 10 µM (IC_90_, 1 h)
Non-mitochondrial respiration	165.5 ± 37.42	167.06 ± 43.96	162.8 ± 39.78
Basal respiration	171.1 ± 21.61	115.10 ± 17.67	57.53 ± 35.25^*^
Maximal respiration	188.7 ± 23.42	130.8 ± 24.15	83.81 ± 39.39^*^
Proton leak	183.7 ± 17.32	140.70 ± 21.47	169.31 ± 24.30
SRC (%)	123.60 ± 22.01	110.5 ± 24.49	92.96 ± 03.64
Coupling efficiency (%)	38.17 ± 15.33	29.93 ± 05.23	07.88 ± 05.64^*^
ATP-linked respiration	49.83 ± 15.83	25.21 ± 16.65^*^	09.89 ± 10.98^**^

Mitochondrial respiration in terms of OCR of log-phase AG83 promastigotes (2 × 10^6^/well) was measured by Seahorse Extracellular Flux Analyzer (XFp) following treatment with Ampho B (100 nM, 1 h, 37 °C) and HePC (10 µM, 1 h, 37 °C), followed by addition of oligomycin (Oligo; 10 µM), FCCP (2 µM), and Rot + AA (1 µM, each), as described in the “Methods” section. Data are expressed as mean (± standard error [SE]) of OCR (pmol/min/2 × 10^6^ parasites) of at least three different experiments in duplicates; ^*^P < 0.05 and ^**^P < 0.01 as compared with control. The parameters of OCR included (i) nonmitochondrial respiration (minimum rate of OCR measurement after Rot + AA injection), (ii) basal respiration (difference between the last OCR measurement before injection of oligomycin and nonmitochondrial respiration), (iii) maximal respiration (difference between maximum OCR after FCCP injection and nonmitochondrial OCR), (iv) proton leak (difference between minimum rate measurement after oligomycin injection and nonmitochondrial respiration), (v) spare respiratory capacity (SRC) (ratio between maximal respiration and basal respiration), (vi) coupling efficiency (ratio between ATP production and basal respiration), and (vii) respiration linked to mitochondrial ATP production (OCR difference before and after oligomycin)

**Table 6:** Effect of conventional antileishmanials (acute response) on mitochondrial oxygen consumption rate (OCR) in murine peritoneal macrophages

Parameters of mitochondrial respiration (pmol/min/10^6^ macrophages)	Control	Ampho B 100 nM	HePC 10 µM
Non-mitochondrial respiration	68.39 ± 28.28	67.16 ± 23.01	75.84 ± 38.63
Basal respiration	41.72 ± 23.21	42.96 ± 23.82	41.50 ± 21.45
Maximal respiration	95.57 ± 35.75	109.90 ± 43.75	82.98 ± 29.80
Proton leak	27.55 ± 11.52	22.76 ± 09.61	29.60 ± 09.48
SRC	176.9 ± 83.97	151.6 ± 66.04	158.3 ± 66.94
Coupling efficiency (%)	68.60 ± 16.84	61.64 ± 17.24	57.51 ± 02.94
ATP-linked respiration	50.82 ± 20.21	52.04 ± 22.86	47.58 ± 20.45
Acute response	0.13 ± 0.17	0.11 ± 0.09	0.09 ± 0.17

Mitochondrial respiration in terms of OCR of murine peritoneal macrophages (1 × 10^6^/well) was measured by Seahorse Extracellular Flux Analyzer (XFp) following treatment with Ampho B (0–100 nM, acute response) and HePC (0–10 µM, acute response) by addition of these drugs in port A followed by addition of oligomycin (Oligo; 10 µM), FCCP (2 µM), and Rot + AA (1 µM, each), as described in the “Methods” section. Data are expressed as mean (± standard error [SE]) of OCR (pmol/min/10^6^ macrophages) of at least three experiments in duplicates

The measurement of OCR in infected peritoneal macrophages was not feasible as cells demonstrated an extremely low baseline value and poor responses to inhibitors of OCR. It has been experimentally proven that profiling of metabolic bioenergetics was dependent on time, sample processing, and longer incubation period [22]. Accordingly, the status of major oxidative phosphorylation regulatory enzymes, cytochrome c oxidase (complex IV or Cox IV) and ATP synthase was measured in *Leishmania*-infected macrophages using ddPCR in terms of copies/µl [9, 23], keeping uninfected murine peritoneal macrophages as the comparator arm.

As compared with uninfected macrophages, infection translated into a significant enhancement of enzymes linked to oxidative phosphorylation, namely *Cox IV* (2.99-fold, *P* < 0.05) and *Atp* *synthase* (4.33-fold, *P* < 0.001) (Fig. 3D(i–ii), E(i–ii); Table 7). Upon addition of antileishmanials at their respective IC_90_ concentrations against amastigotes, Ampho B (50 nM) and HePC (5 µM) significantly downregulated *Cox IV* and *Atp synthases*; Ampho B (50 nM) induced a 1.94-fold and 2.84-fold (*P* < 0.05) depletion in *Cox IV* and *tp* *synthase*, respectively; similarly, HePC (5 µM) curtailed the expression of *Cox IV* and *Atp* *synthase* by 3.21-fold (*P* < 0.05) and 4.33-fold (*P* < 0.01), respectively in comparison to infected macrophages. In sum, the proportion of inhibition by HePC was significantly greater than by Ampho B (Fig. 3D(i–ii), E(i–ii); Table 7).

**Table 7:** Effect of conventional antileishmanials upon oxidative phosphorylation regulatory genes in *L. donovani*-infected macrophages

Target genes (copies/µl)	Uninfected Mϕ	Infected Mϕ	Ampho B 50 nM	HePC 5 µM
*Cox IV*	0.56 ± 0.18	1.67 ± 0.33^*^	0.86 ± 0.16	0.52 ± 0.09^#^
*Atp synthase*	0.46 ± 0.13	1.99 ± 0.37^***^	0.70 ± 0.11^#^	0.46 ± 0.07^##^
*Pdk1*	1.23 ± 0.15	1.78 ± 0.32	1.58 ± 0.14	1.01 ± 0.05
*Ldh*	1.26 ± 0.42	1.83 ± 0.33	1.57 ± 0.49	1.16 ± 0.41

Effect of Ampho B (50 nM, 48 h, 37 °C) and HePC (5 µM, 48 h, 37 °C) on the expression of oxidative phosphorylation regulatory genes of mitochondrial ETC in terms of copies/µL was measured by ddPCR using target-specific primers in *L. donovani* (AG83)-infected murine peritoneal macrophages, as described in the “Methods” section. Data are expressed as mean (± standard error [SE]) of at least three different experiments in duplicates; ^*^P < 0.05, ^**^P < 0.01, and ^*^P < 0.001 as compared with uninfected macrophages and ^#^*P* < 0.05 and ^##^*P* < 0.01 as compared with infected macrophages

Expression of *pyruvate dehydrogenase kinase 1* (*Pdk1*) and *lactate dehydrogenase* (*Ldh*) can identify whether the metabolic driving force is toward oxidative phosphorylation or glycolysis. As pyruvate is derived from glycolysis, it either enters into the mitochondrial tricarboxylic acid (TCA) cycle, where it is negatively regulated by *Pdk1*, or is reduced to lactate by *Ldh* via the anaerobic metabolic pathway. Infection caused a marginal enhancement in *Pdk1* (1.44-fold) and *Ldh* (1.45-fold (Fig. 3D(iii), D(iv), E(iii), E(iv); Table 7), indicating that the cellular metabolic drive is strongly tilted toward oxidative phosphorylation. However, Ampho B and HePC failed to have an impact on *Pdk1* or *Ldh* (Fig. 3D(iii), D(iv), E(iii), E(iv); Table 7).

### Effect of Ampho B and HePC upon mitochondrial substrate utilization

The mitochondrial TCA cycle can potentially acquire its building block, acetyl-CoA, via three metabolic pathways: (i) following amino acid catabolism, (ii) β-oxidation of fatty acids, and/or (iii) glycolysis, following oxidative decarboxylation of pyruvate [18]. To identify the major pathway(s) adopted by promastigotes, a substrate oxidation assay was performed, using specific inhibitors, namely: (i) BPTES (inhibition of glutamine through glutaminase 1), (ii) etomoxir (long-chain fatty acids (LCFAs) through inhibition of carnitine palmitoyl transferase 1a (CPT1a), and (iii) UK5099 (pyruvate through inhibition of the mitochondrial pyruvate carrier (MPC).

**Fig. 4:**
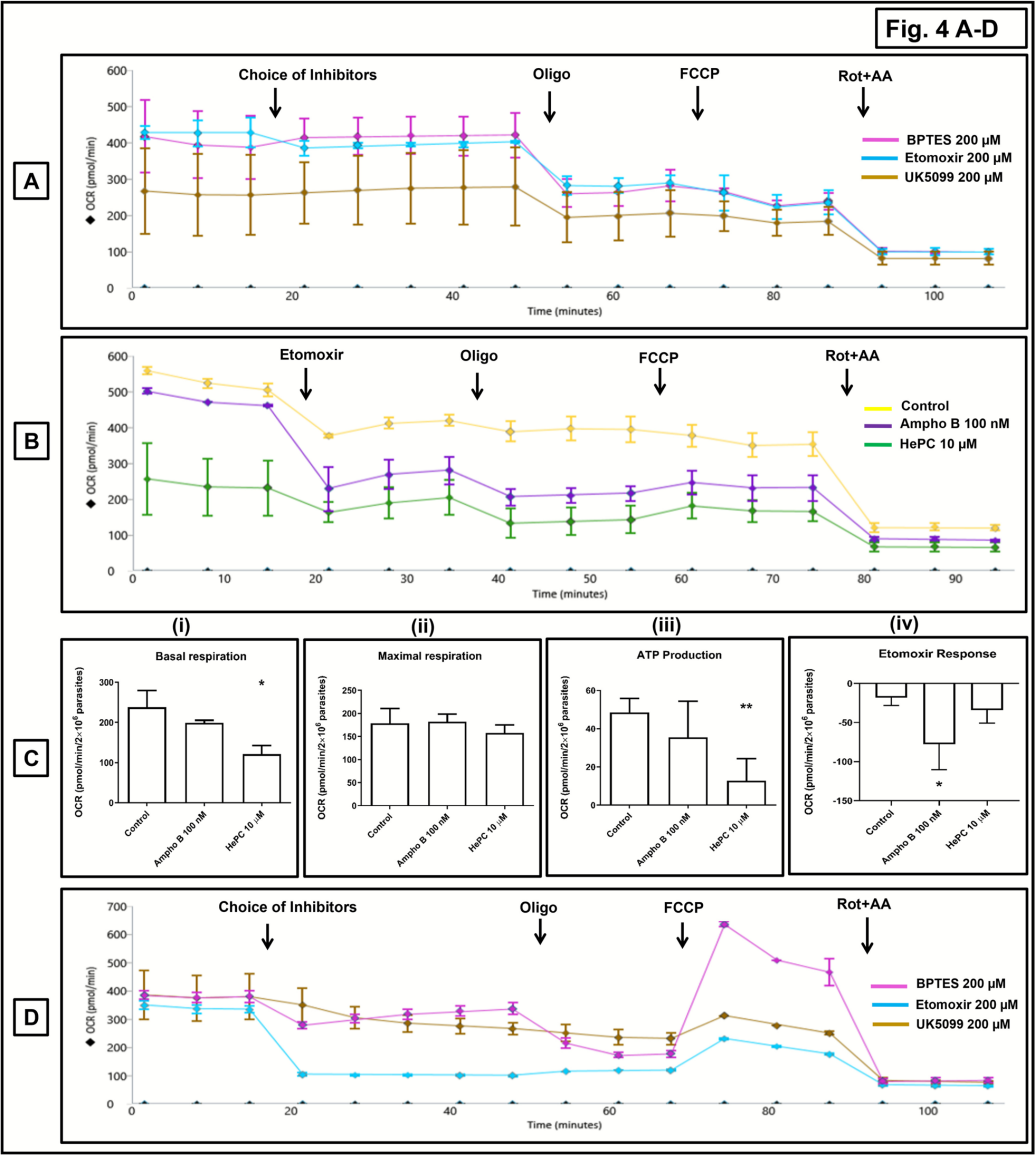
Assessment of substrate oxidation stress test in *Leishmania donovani* promastigotes treated with conventional antileishmanials. **A** Representative profile of substrate oxidation by mitochondrial tricarboxylic acid (TCA) cycle of at least three independent experiments of log-phase *Leishmania donovani* promastigotes (AG83, 2 × 10^6^ cells/well) treated with BPTES (200 µM), Etomoxir (200 µM), or UK5099 (200 µM) in port A followed by addition of oligomycin (Oligo; 10 µM), FCCP (2 µM), and Rot + AA (1 µM, each), as measured by Seahorse Extracellular Flux Analyzer (XFp). **B** Representative profile of mitochondrial respiration (OCR) of at least three independent experiments of log-phase promastigotes (AG83, 2 × 10^6^ cells/well) treated with Ampho B (100 nM, 1 h, 37 °C) or HePC (10 µM, 1 h, 37 °C), following addition of etomoxir (200 µM), oligomycin (Oligo; 10 µM), FCCP (2 µM), and Rot + AA (1 µM, each), as measured by Seahorse Extracellular Flux Analyzer (XFp). **C** Bar graphs of (**i**) basal respiration, (**ii**) maximal respiration, (**iii**) ATP production, and (**iv**) acute response of Etomoxir. Data are expressed as the mean (± standard error [SE]) of OCR (mpH/min/2 × 10^6^ parasites) of at least three experiments in duplicates; ^*^P < 0.05 and ^**^P < 0.01 as compared with untreated parasites. **D** Representative profile of substrate oxidation by mitochondrial tricarboxylic acid (TCA) cycle of at least three independent experiments of murine peritoneal macrophages (1 × 10^6^ cells/well) treated with BPTES (200 µM), Etomoxir (200 µM), or UK5099 (200 µM) in port A followed by addition of oligomycin (Oligo; 10 µM), FCCP (2 µM), and Rot + AA (1 µM, each), as measured by Seahorse Extracellular Flux Analyzer (XFp)

Etomoxir (200 µM) diminished the OCR (Fig. 4A), whereas BPTES (200 µM) and UK5099 (200 µM) failed to impair the OCR (Fig. 4A), indicating that promastigotes primarily acquire acetyl CoA for the TCA cycle via β oxidation of fatty acids. The suppression of OCR in presence of etomoxir (in terms of acute response) was greater by Ampho B (100 nM, 1 h) than HePC (10 µM, 1 h), indicating that the inhibition of mitochondrial fatty acid oxidation by Ampho B was significantly greater than by HePC (Fig. 4B, C(iv); Table 8). However, the overall depletion of different parameters of mitochondrial respiration, namely basal respiration or maximal respiration or coupling efficiency was consistently higher by HePC than Ampho B (Fig. 4B, C(i–iii); Table 8). The inhibitors of mitochondrial TCA cycle substrate oxidation, BPTES, etomoxir, and UK5099, substantially reduced the OCR in macrophages, indicating that the mechanism/pathway of mitochondrial TCA cycle substrate oxidation in host macrophages differs from *Leishmania* promastigotes (Fig. 4D).

**Table 8:** Effect of conventional antileishmanials on mitochondrial substrate utilization assay in *L. donovani* promastigotes

Parameters of mitochondrial respiration (pmol/min/2 × 10^6^ parasites)	Control	Ampho B 100 nM (IC_90_, 1 h)	HePC 10 µM (IC_90_, 1 h)
Non-mitochondrial respiration	90.61 ± 20.47	100.40 ± 13.77	113.60 ± 24.45
Basal respiration	237.8 ± 41.67	199.20 ± 06.08	121.40 ± 21.07^*^
Maximal respiration	178.90 ± 31.61	181.9 ± 16.41	157.90 ± 17.01
Proton leak	152.50 ± 49.15	132.10 ± 12.88	119.80 ± 07.41
SRC (%)	77.48 ± 05.45	78.19 ± 12.56	79.72 ± 12.69
Coupling efficiency (%)	33.01 ± 07.10	19.06 ± 06.37	01.23 ± 07.98^**^
ATP-linked respiration	48.45 ± 07.45	35.48 ± 18.94	12.89 ± 11.45^**^
Acute response of etomoxir	−18.34 ± 9.66	−77.68 ± 32.61^*^	−34.08 ± 16.50

Effect of Ampho B (100 nM, 1 h, 37 °C) and HePC (10 µM, 1 h, 37 °C) upon mitochondrial respiration in terms of OCR of log-phase AG83 promastigotes (2 × 10^6^/well) was measured by Seahorse Extracellular Flux Analyzer (XFp) with addition of Etomoxir (200 µM) in port A followed by addition of oligomycin (Oligo; 10 µM), FCCP (2 µM), and Rot + AA (1 µM, each), as described in the “Methods” section. Data are expressed as mean (± standard error [SE]) of OCR (pmol/min/2 × 10^6^ parasites) of at least three different experiments in duplicates; ^*^P < 0.05 and ^**^P < 0.01 as compared with control

### Effect of Ampho B and HePC upon glycolytic functions

**Fig. 5:**
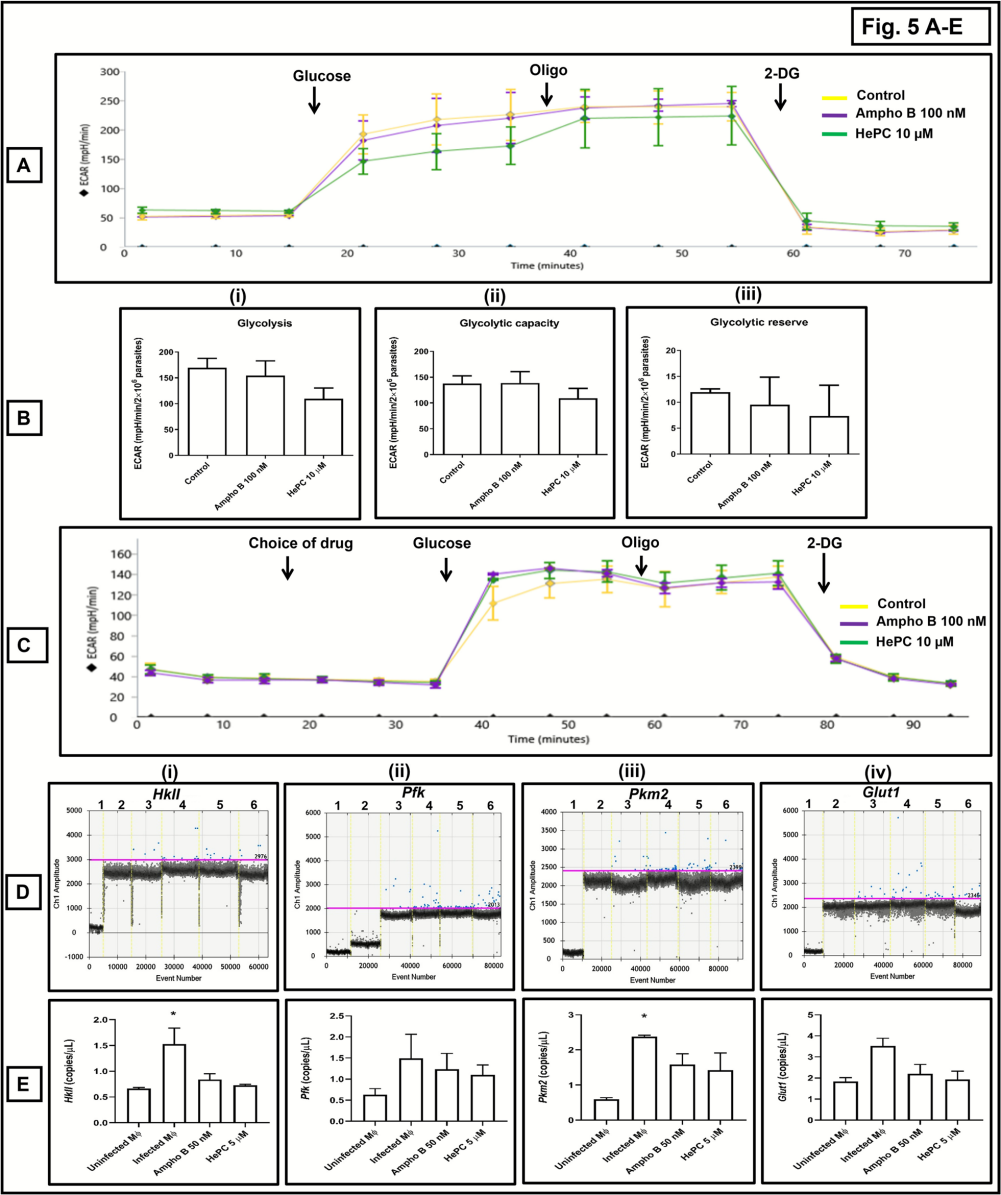
Assessment of glycolytic functions in *Leishmania donovani* treated with conventional antileishmanials. **A** Representative profile of glycolytic activities (ECAR) of at least three different experiments of log-phase promastigotes (AG83, 2 × 10^6^ cells/well) following treatment with Ampho B (100 nM, 1 h, 37 °C) or HePC 10 µM, 1 h, 37 °C) and measured by Seahorse Extracellular Flux Analyzer (XFp) following addition of glucose (10 mM), oligomycin (Oligo; 10 µM), and 2-DG (50 mM), as described in the “Methods” section. **B** Bar graphs of (**i**) glycolysis, (**ii**) glycolytic capacity, and (**iii**) glycolytic reserve. Data are expressed as the mean (± standard error [SE]) of ECAR (mpH/min/2 × 10^6^ parasites) of at least three experiments in duplicates. **C** Representative profile of glycolytic activities (ECAR) of at least three different experiments of murine peritoneal macrophages (1 × 10^6^ cells/well) following treatment with Ampho B (100 nM) or HePC (10 µM) in port A (acute response) and measured by Seahorse Extracellular Flux Analyzer (XFp) following addition of glucose (10 mM), oligomycin (Oligo; 10 µM) and 2-DG (50 mM) as described in the “Methods” section. **D** Representative profiles of genes related to glycolytic functions by absolute quantification by ddPCR, namely (**i**) *HkII*, (**ii**) *Pfk*, (**iii**) *Pkm2*, and (**iv**) *Glut1* in AG83-infected peritoneal macrophages incubated with Ampho B or HePC as described in the “Methods” section. One-dimensional plot of droplets measured for fluorescence signals (amplitude indicated on *y*-axis) emitted from respective target-specific primers; lane 1: negative control (NC; nuclease-free water), lane 2: nontemplate controls (NTC), lane 3: uninfected peritoneal macrophages, lane 4: infected peritoneal macrophages treated with Ampho B (lane 5) or HePC (lane 6). EvaGreen-bound positive droplets are shown in blue, while negative droplets are shown in black, with expression of the genes quantified as copies/μl as described in the “Methods” section. **E** Bar graphs showing (**i**) *HkII*, (**ii**) *Pfk*, (**iii**) *Pkm2*, and (**iv**) *Glut1* in copies/μl in macrophages following infection and after treatment with Ampho B or HePC. Data are expressed as the mean (± standard error [SE]) of at least three experiments in duplicates; ^*^P < 0.05 as compared with uninfected macrophages

With regard to glycolysis in *L. donovani* promastigotes, Ampho B (100 nM, 1 h) did not alter glycolytic functions (glycolysis or glycolytic capacity or glycolytic reserve) (Fig. 5A, B; Table 9). However, HePC (10 µM, 1 h) caused a 35.49% inhibition in glycolysis and a 21.02% inhibition in glycolytic capacity, which translated into a 38.82% inhibition in the glycolytic reserve (Fig. 5A, B(i–iii); Table 9). The acute responses with Ampho B and HePC were evaluated by directly adding the drugs in port A of XFp cartridges, wherein neither Ampho B nor HePC altered the ECAR (Supplementary Fig. 2C, D; Supplementary Table 2B). In host macrophages, Ampho B (100 nM) and HePC (10 μM) did not alter the glycolytic activities (Fig. 5C; Table 10).

**Table 9:** Effect of conventional antileishmanials on extracellular acidification rate (ECAR) in *L. donovani* promastigotes

Parameters of glycolytic activities (mpH/min/2 × 10^6^ parasites)	Control	Ampho B 100 nM (IC_90_, 1 h)	HePC 10 µM (IC_90_, 1 h)
Non-glycolytic acidification	52.44 ± 06.11	55.34 ± 09.90	47.48 ± 07.71
Glycolysis	169.6 ± 18.13	154.3 ± 28.53	109.4 ± 20.83
Glycolytic capacity	137.9 ± 14.49	138.8 ± 22.19	108.9 ± 19.58
Glycolytic reserve	11.95 ± 0.61	09.56 ± 05.32	07.31 ± 5.99

The effect of Ampho B (100 nM, 1 h, 37 °C) and HePC (10 µM, 1 h, 37 °C) upon the ECAR of log-phase AG83 promastigotes (2 × 10^6^/well) was measured in a Seahorse Extracellular Flux Analyzer (XFp) following the addition of glucose (10 mM), oligomycin (Oligo; 10 µM), and 2-DG (50 mM), as described in the “Methods” section. Data are expressed as mean (± standard error [SE]) of ECAR (mpH/min/2 × 10^6^ parasites) of at least three different experiments in duplicates. The parameters of ECAR included (i) nonglycolytic acidification (last ECAR rate measurement prior to glucose injection), (ii) glycolysis (difference between maximum ECAR after glucose injection and nonglycolytic acidification), (iii) glycolytic capacity (difference between maximum ECAR after oligomycin injection and nonglycolytic acidification), and (iv) glycolytic reserve (difference between basal ECAR or glycolysis from glycolytic capacity)

**Table 10:** Effect of conventional antileishmanials (acute response) on extracellular acidification rate (ECAR) of peritoneal macrophages

Parameters of glycolytic activities (mpH/min/10^6^ macrophages)	Control	Ampho B 100 nM	HePC 10 µM
Non-glycolytic acidification	23.77 ± 08.14	24.93 ± 07.11	24.32 ± 06.18
Glycolysis	51.48 ± 11.48	47.77 ± 17.45	51.21 ± 15.76
Glycolytic capacity	50.74 ± 12.71	43.40 ± 15.11	45.21 ± 16.32
Glycolytic reserve (%)	92.70 ± 05.68	85.67 ± 05.49	78.33 ± 04.27
Acute response	04.69 ± 03.90	04.43 ± 04.39	04.21 ± 03.13

The effect of Ampho B (0–100 nM, acute response) and HePC (0–10 µM, acute response) upon the ECAR of murine peritoneal macrophages (1 × 10^6^/well) was measured in a Seahorse Extracellular Flux Analyzer (XFp) by addition of these drugs in port A followed by the addition of glucose (10 mM), oligomycin (Oligo; 10 µM), and 2-DG (50 mM), as described in the “Methods” section. Data are expressed as mean (± standard error [SE]) of ECAR (mpH/min/10^6^ macrophages) of at least three experiments in duplicates

The transcriptional status of glycolysis rate-limiting enzymes, namely *hexokinase II* (*HkII*), *phosphofructokinase* (*Pfk*), *pyruvate kinase* *M2 *(*Pkm2*), and glucose transporter *Glut1* (primary glucose transporter of macrophages) was assessed in *Leishmania*-infected macrophages, keeping uninfected murine peritoneal macrophages as the comparator arm. There was an enhancement of glycolytic enzymes, *HkII* (2.32-fold, *P* < 0.05), *Pkm2* (4.03-fold, *P* < 0.05), *Pfk* (2.36- fold), and *Glut1* (1.93- fold) (Fig. 5D, E; Table 11). However, Ampho B and HePC did not exert an impact on these glycolytic enzymes (Fig. 5D, E; Table 11).

**Table 11:** Effect of conventional antileishmanials upon glycolysis regulatory genes in *L. donovani*-infected macrophages

Target genes (copies/µl)	Uninfected Mϕ	Infected Mϕ	Ampho B 50 nM	HePC 5 µM
*HkII*	0.66 ± 0.02	1.53 ± 0.31^*^	0.84 ± 0.11	0.73 ± 0.02
*Pfk*	0.63 ± 0.14	1.49 ± 0.58	1.24 ± 0.37	1.10 ± 0.23
*Pkm2*	0.59 ± 0.05	2.38 ± 0.37^*^	1.58 ± 0.31	1.42 ± 0.49
*Glut1*	1.83 ± 0.18	3.53 ± 0.36	2.20 ± 0.44	1.93 ± 0.38

Effect of Ampho B (50 nM, 48 h, 37 °C) and HePC (5 µM, 48 h, 37 °C) on the expression of glycolysis regulatory genes in terms of copies/µL was measured by ddPCR using target-specific primers in *L. donovani* (AG83)-infected murine peritoneal macrophages, as described in the “Methods” section. Data are expressed as mean (± standard error [SE]) of at least three different experiments in duplicates; ^*^P < 0.05 as compared with uninfected macrophages

### Impact of *L. donovani* infection on AMPK signaling pathway

The metabolic interplay between parasites and host cells is critical for progression of infection [11, 24], and this metabolic rewiring is regulated by AMPK, a central regulator of metabolic bioenergetics. Cells produce ATP via two major energy producing pathways, namely, oxidative phosphorylation and glycolysis, and AMPK is tightly regulated by the cellular AMP/ATP ratio [9, 24]. Thus, the transcriptional status of major regulatory molecules of the AMPK axis was assessed in *Leishmania*-infected macrophages, namely, *Ampk*, *Lkb1*, and *Sirt1*, along with the mitochondrial biogenesis marker, *Pgc1α*, and *Mtor*, a crucial metabolic sensor, keeping uninfected murine peritoneal macrophages as the comparator arm.

**Fig. 6:**
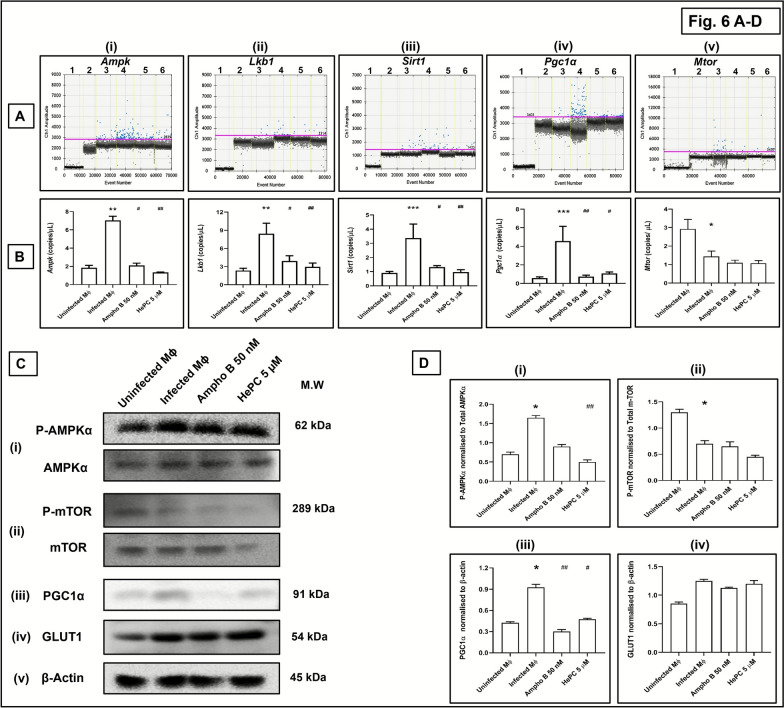
Impact of conventional antileishmanials on AMPK signaling pathway. **A** Status of AMPK axis in *Leishmania*-infected peritoneal macrophages incubated with Ampho B (50 nM, 48 h, 37 °C) or HePC (5 µM, 48 h, 37 °C) as described in the “Methods” section. Representative profiles of genes, namely, (**i**) *Ampk*, (**ii**)*Lkb1*, (**iii**) *Sirt1*, and (**iv**) *Pgc1α*, as quantified by ddPCR. One-dimensional plot of droplets measured for fluorescence signals (amplitude indicated on *y*-axis) emitted from respective target-specific primers; lane 1: negative control (NC; nuclease-free water), lane 2: nontemplate controls (NTC), lane 3: uninfected peritoneal macrophages, lane 4: infected peritoneal macrophages treated with Ampho B (lane 5) or HePC (lane 6). EvaGreen-bound positive droplets are shown in blue, while negative droplets are shown in black, with expression of genes quantified as copies/μl as described in the “Methods” section. **B** Bar graphs showing copies/μl of (**i**) *Ampk*, (**ii**) *Lkb1*, (**iii**) *Sirt1*, and (**iv**) *Pgc1α* in uninfected macrophages and infected peritoneal macrophages treated with Ampho B or HePC. Data are expressed as the mean (± standard error [SE]) of at least three experiments in duplicates; ^*^P < 0.05, ^**^P < 0.01, and ^***^P < 0.001 as compared with uninfected macrophages; ^*#*^*P* < 0.05 and ^##^*P* < 0.01 as compared with infected macrophages. **C** Representative profile of *L. donovani*-infected peritoneal macrophages treated with Ampho B or HePC as described in the “Methods” section. Cell lysates were subjected to immune blotting and probed with antibodies against (**i**) AMPKα and (**ii**) mTOR, along with their phosphorylated counterparts, along with (**iii**) PGC1α, (**iv**) GLUT1 , and (**v**) β-actin as described in the “Methods” section. **D** Bar graphs showing relative expression of (**i**) AMPKα, (**ii**) mTOR, (**iii**)PGC1α, and (**iv**) GLUT1 in terms of arbitrary units in uninfected and infected peritoneal macrophages treated with Ampho B or HePC. Phosphorylated proteins were normalized with respect to their respective total protein, whereas PGC1α and GLUT1 were normalized with β-actin. Data are expressed as the mean (± standard error [SE]) of at least three experiments; ^*^P < 0.05 as compared with uninfected macrophages; ^#^*P* < 0.05 and ^##^*P* < 0.01 as compared with infected macrophages

As compared with uninfected macrophages, infection translated into a significant increase in *Ampk* (3.82-fold, *P* < 0.01), *Lkb1* (3.59-fold, *P* < 0.01), *Sirt1* (3.70-fold, *P* < 0.001), and *Pgc1*α (8.0-fold, *P* < 0.001**);** whereas *Mtor* was downregulated by 2.03-fold (*P* < 0.05; Fig. 6A, B; Table 12). This was reversed by the addition of Ampho B and HePC (at the respective IC_90_ concentration against amastigotes), as they both caused a significant downregulation of the AMPK axis (*Ampk*, *Lkb1*, *Sirt1*) and *Pgc1α*, except *Mtor*, as compared with infected macrophages. Ampho B (50 nM) diminished the expression of *Ampk*, *Lkb1*, *Sirt1* and *Pgc1α* by 3.35-fold (*P* < 0.05), 2.16-fold (*P* < 0.05), 2.57-fold (*P* < 0.05), and 6.16-fold (*P* < 0.01) respectively, whereas HePC (5 µM) decreased the expression of *Ampk*, *Lkb1*, *Sirt1*, and *Pgc1α* by 5.28-fold (*P* < 0.01), 2.85-fold (*P* < 0.01), 3.47-fold (*P* < 0.01), and 4.26-fold (*P* < 0.05), respectively, as compared with infected macrophages; hence, the proportion of inhibition by HePC was significantly greater than by Ampho B, a notable exception being *Pgc1α* (Fig. 6A, B; Table 12); furthermore, HePC negligibly curtailed the expression of *Mtor* (Fig. 6A, B; Table 12).

**Table 12:** Effect of conventional antileishmanials upon major metabolism regulatory genes in *L. donovani*-infected macrophages

Target genes (copies/µl)	Uninfected Mϕ	Infected Mϕ	Ampho B 50 nM	HePC 5 µM
*Ampk*	1.84 ± 0.26	7.03 ± 0.45^**^	2.10 ± 0.25^#^	1.33 ± 0.05^##^
*Lkb1*	2.35 ± 0.39	8.44 ± 1.71^**^	3.91 ± 0.88^#^	2.96 ± 0.65^##^
*Sirt1*	0.91 ± 0.10	3.37 ± 0.99^***^	1.31 ± 0.11^#^	0.97 ± 0.17^##^
*Pgc1α*	0.57 ± 0.14	4.56 ± 1.60^***^	0.74 ± 0.14^##^	1.07 ± 0.17^#^
*Mtor*	2.92 ± 0.51	1.44 ± 0.29^*^	1.1 ± 0.14	1.07 ± 0.15

Effect of Ampho B (50 nM, 48 h, 37 °C) and HePC (5 µM, 48 h, 37 °C) upon a few major metabolism regulatory gene expressions in terms of copies/µL was measured by ddPCR using target-specific primers in *L. donovani* (AG83)-infected murine peritoneal macrophages, as described in the “Methods” section. Data are expressed as mean (± standard error [SE]) of at least three different experiments in duplicates; ^*^P < 0.05, ^**^P < 0.01, and ^*^P < 0.001 as compared with uninfected macrophages and ^#^*P* < 0.05 and ^##^*P* < 0.01 as compared with infected macrophages

To further understand regulation of metabolic adaptations following establishment of infection, status of the AMPK signalling pathway was analyzed in presence of Ampho B and HePC at a protein level. Following infection, a significant increase in the expression of phosphorylated component of AMPK (P-AMPK, *P* < 0.05) and PGC1α (*P* < 0.05) was evident (Fig. 6C(i), (iii) and D(i), (iii)), which was decreased by Ampho B and HePC. The degree of inhibition by HePC was significantly greater than by Ampho B (Fig. 6C(i), D(i)). Infection significantly downregulated the expression of P-mTOR (*P* < 0.05) (Fig. 6C(ii), D(ii)), whereas expression of GLUT1 marginally increased (Fig. 6C(iv), D(iv)). HePC marginally diminished the expression of mTOR but had no impact on GLUT1 (Fig. 6C(ii), (iv) and D(ii), (iv)).

## Discussion

As leishmaniasis is an NTD, repurposing and reevaluation by “on-target” (i.e., exploring newer options for a drug different from the originally known target) and “off-target” (i.e., establishing indications for a molecule that mediates its action via an unanticipated or novel target) approaches is a viable option for ensuring a sustainable pipeline of antileishmanials [25, 26]. In fact, all drugs active against leishmaniasis have emerged following repurposing and demonstrate multiple mode(s) of action [25, 27].

Ampho B, a macrolide polyene, binds and form complexes with membrane ergosterols and causes pore formation into ion channels that translate into increased membrane permeability and cellular death [1, 28]. As *Leishmania* parasites have a deficient antioxidant system, the impact of Ampho B, if any, on the redox system remains open ended [1, 27]. However, HePC, being amphiphilic in nature, incorporates into the cell plasma membrane, targets phosphocholine cytidylyltransferase, inhibits biosynthesis of phosphatidylcholine, and hampers cell survival [27]. In vitro studies have confirmed that HePC causes apoptosis in a “caspase-3-dependent” manner, as endorsed in promastigotes of *L. amazonensis*, *L. infantum*, and *L. donovani*, wherein HePC, by targeting the mitochondrion, generated ROS, caused membrane depolarization, inhibited cytochrome c oxidase activity, and affected the homeostasis of Ca^2+^, culminating in an apoptosis-like death [27, 29]. HePC also has immunomodulatory functions as it restores the Th1/Th2 balance by increasing host expression of nitric oxide synthase 2 (iNOS2) [1, 30]. Despite a considerable amount of information available concerning the mode of action of HePC, detailed insight(s) regarding its potential modulation of mitochondrial bioenergetics remains to be established; similarly, the impact of Ampho B upon similar parameters remains open ended. Accordingly, this study focused on establishing whether conventional antileishmanials, Ampho B and HePC, by targeting the mitochondrion, modulate the cellular bioenergetics of *Leishmania donovani* promastigotes and host macrophages.

Disruption of the redox homeostasis is an established modality exerted by antileishmanials [13], and was endorsed in this study by the increased oxidative burst (Fig. 1A, B; Table 2), along with an increased generation of mitochondrial superoxide (Fig. 2A; Table 3). Although Ampho B induced oxidative stress, the proportion of generation of free radicals was consistently lower than HePC (Figs. 1A and 2A; Tables 2 and 3), corroborating that HePC mediated its leishmanicidal activity primarily via a redox imbalance, whereas Ampho B possibly utilized other modalities, induction of oxidative stress being a relatively lower contributory factor. In antimony and Ampho B-resistant *Leishmania* strains, higher levels of intracellular thiols have been reported [28, 31]. However, the impact of Ampho B, if any, on mitochondrial generation of superoxide and the mitochondrial ETC, had not been determined, and in a head-to-head comparison with HePC, at their respective IC_50_, demonstrated inhibition of mitochondrial functions, the degree of inhibition being consistently higher with HePC (Fig. 2A–C, Tables 3, 4).

Despite the growing evidence of the mitochondrial ETC being an attractive drug target, there exists a knowledge gap regarding mitochondrial-based energy metabolism in *Leishmania* parasites [9]. Both drugs inhibited mitochondrial respiration of *L. donovani* promastigotes, in terms of basal respiration, maximal respiration, proton leak, and respiration linked to ATP production, with the proportion of inhibition by HePC consistently being higher (Fig. 3A, B; Table 5). Studies have indicated that HePC at higher concentrations (25 µM) inhibited mitochondrial respiration upon prolonged/overnight incubation using Clark’s oxygen electrode [29], but this study firmly established that the mitochondrial inhibitory action of HePC occurred immediately and at lower concentrations. As *Leishmania* parasites lack the spare respiratory capacity (SRC) [13, 32], the addition of FCCP failed to increase the OCR (Fig. 3A). Furthermore, the β-oxidation of fatty acids of mitochondrial TCA cycle was established as the major building block of acetyl CoA, the key fuel for the TCA cycle (Fig. 4A), and its inhibition by Ampho B being greater than by HePC (Fig. 4B, C; Table 8). This endorsed previous reports wherein fatty acid oxidation was reported as the key source of nutrients for intracellular amastigotes in *Leishmania mexicana* [33]. Additionally, in *Trypanosoma cruzi*, causative agent of Chagas disease, alteration of β-oxidation of fatty acids inhibited their intracellular growth [34]. Furthermore, the lipid composition in Ampho B-resistant strains of *L. donovani* promastigotes demonstrated a significantly elevated percentage of saturated fatty acids [35], indicative of a crucial role of fatty acid metabolism in mediating their leishmanicidal effect. Thus, chemotherapeutic interventions of the fatty acid oxidation pathway of *L. donovani* promastigotes by conventional antileishmanials could be evaluated as a therapeutic option.

The depletion of ATP in *Leishmania* is not limited to disruption of mitochondrial functions, but can also occur following inhibition of glycolysis, as for example, ascaridole, a potential antileishmanial endoperoxide, that failed to have an impact on mitochondrial functions, but inhibited glycolytic functions caused ATP depletion and membrane depolarization [13, 36]. Accordingly, as glycolysis and the mitochondrial ETC are two major sources of cellular ATP, the impact of Ampho B and HePC upon glycolytic functions, mitochondrial oxygen consumption, and substrate utilization was assessed wherein both compounds demonstrated a limited impact on glycolysis (Fig. 5A, B; Table 9).

A promising avenue emerging as for the treatment of bacterial, viral, and parasitic diseases are host-directed therapies that targets pathogens by acting directly on host molecules that are redundant for the host, but critical for the pathogen, thus augmenting the safety index and narrowing the chance of developing resistance [34, 37]. The availability of genomics, proteomics, and computational biology has allowed for the identification of several host pathways that hold promise as targets for host-directed therapies. Targeting the host metabolic machinery is one of them [36, 37] a classical example being the AMPK–SIRT1–mTOR axis that, by acting as a metabolic sensor and regulator of cells, modulates a cascade of complex and multifaceted events that are associated with cell phenotype, cell functionality, energy production, and cytokine release [2, 24]. AMPK, a fuel-sensing molecule, becomes activated following shortage of energy, causing an increased AMP/ATP ratio. Two upstream kinases, serine threonine liver kinase B1 (LKB1) and SIRT1 (NAD^+^-dependent histone deacetylases) can activate AMPK by phosphorylating a threonine residue (Thr172) on its catalytic α-subunit [9, 38]. Activated AMPK also conserves energy by globally reducing synthesis of proteins via antagonizing mTOR, a kinase necessary for formation of the elF4F complex, critical for initiation of translation [39]. Taken together, AMPK, LKB1, SIRT1, and mTOR are key regulators of the cellular metabolic bioenergetics [24, 39].

In cases of malaria, hepatic cells infected by *Plasmodium* exhibited decreased activation of AMPK, and this translated into a reduced parasite burden [40]; on the contrary, *L. infantum* activated AMPK in infected macrophages [9], and cleavage of mTOR supported parasite survival [41]. However, based on the causative *Leishmania* species, host–pathogen interactions, and its outcome in-terms of disease pathogenesis, considerable variation has been reported [42]; therefore, information derived from one *Leishmania* species can often not be extrapolated to other species. Dissecting the metabolic profiles in trypanosomatids is still in its infancy, and comprehensive understanding of host metabolic bioenergetics is needed [34, 37].

Leishmaniasis is associated with M2 polarization of macrophages that facilitates parasite survival [43]. For their energy requirement, parasites are known to utilize fatty acid oxidation, by inducing transcription of PGC-1α along with heightened mitochondrial oxidative phosphorylation and enhanced AMPK expression [7, 8, 11, 21]. This was corroborated by the transcriptomic analysis of *L. donovani*-infected (48 h) macrophages versus uninfected macrophages, where there was a significant upregulation of several oxidative phosphorylation regulatory genes (*Cox IV*, *Atp synthase*) and the *Ampk–Lkb1–Sirt1* axis (Figs. 3D, E and 6A, B). Both Ampho B and HePC downregulated the expression of *Ampk–Lkb1–Sirt1* axis, along with inhibition of oxidative phosphorylation regulatory genes (Figs. 3D, E and6A, B); the inhibition of HePC was greater than Ampho B. A notable exception was *Pgc1α* (Fig. 6A, B), possibly because it not only regulates mitochondrial biogenesis but is also linked with fatty acid oxidation of mitochondria [44], the primary source of Acetyl Co-A in *L. donovani* (Fig. 4). Taken together, conventional antileishmanials mediate reversal of the M2 polarized state of infected macrophages, by attenuating the mitochondrial metabolism, leading to a reduced parasite burden.

In infected macrophages, the transcriptional expression of several regulatory enzymes of glycolysis, likely, *HkII*, *Pfk*, *Pkm2*, and glucose transporter *Glut1* were marginally upregulated (Fig. 3D, E), but not as substantially as the oxidative phosphorylation regulatory molecules (Fig. 5D, E). Additionally, as Ampho B and HePC failed to have an impact on the glycolytic flux-associated regulatory enzymes, it corroborated that their antileishmanial potential is primarily via inhibition of mitochondrial energy metabolism (Fig. 5D, E). Translational-level studies in macrophages infected by *L. donovani* also complemented these findings (Fig. 6C, D). The suppressed mTOR activity in infected macrophages is possibly secondary to the counter-regulatory phenomenon of AMPK. Additionally, mTOR activity promotes the glycolytic flux [24].

## Conclusions

**Fig. 7:**
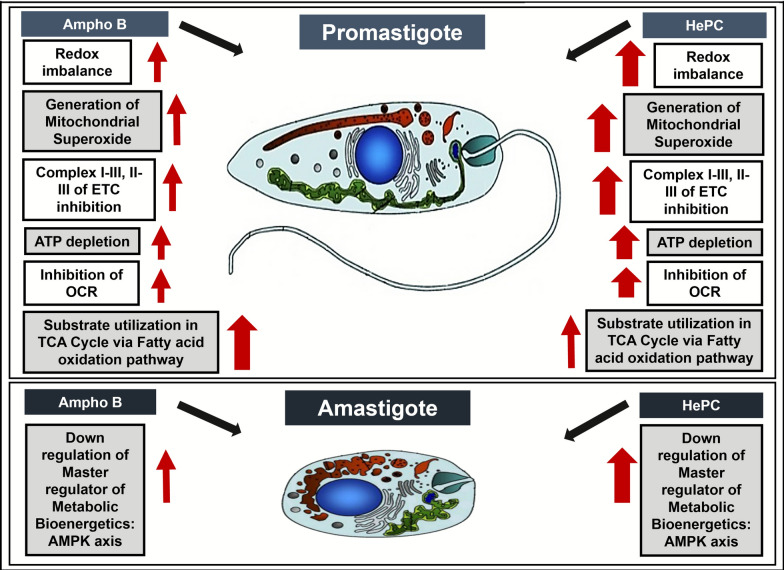
Reprogramming of metabolic bioenergetics by *Leishmania* *donovani* and the impact of conventional antileishmanials Ampho B and HePC. HePC and Ampho B both exert noxious effects on *Leishmania donovani* parasites by inducing dysfunction of parasite mitochondria substantially and modulating metabolic bioenergetics in favor of parasite clearance, with impact at their respective IC_50_–IC_90_ doses, being higher with HePC than Ampho B

This study aimed at bridging the knowledge gap regarding underlying mechanism(s) contributing toward the leishmanicidal activity of conventional antileishmanials Ampho B and HePC, using *in vitro *and *ex vivo* models. *Leishmania* parasites, being digenetic kinetoplastid protozoa, have a single mitochondrion, which is one of the vital sites of generation of ROS and regulation of cellular metabolic bioenergetics, thus making this organelle a potential target for antileishmanial chemotherapeutics. Flow cytometry and spectrophotometric studies in *L. donovani* promastigotes established that Ampho B and HePC target the mitochondria, as evident by increased generation of superoxide along with inhibition of enzymatic activities of complex I–III and II–III of mitochondrial ETC. Furthermore, bioenergetics studies validated that Ampho B and HePC caused a significant inhibition of mitochondrial oxygen consumption, but their impact on glycolysis was negligible. The proportion of mitochondrial dysfunction by HePC exceeded Ampho B. *Leishmania* amastigotes reside and replicate within mammalian macrophages, and devise strategies to manipulate the macrophage metabolic status in favor of their survival included hijacking the AMPK–SIRT1–mTOR pathway; hence, this immune–metabolic pathway could serve as a novel intervention. As compared with uninfected macrophages, when challenged with *L. donovani* promastigotes, they demonstrated a substantially elevated expression of AMPK, LKB1, and SIRT1, along with oxidative phosphorylation regulatory molecules, COX IV, ATP synthase, and a diminished expression of mTOR. Ampho B and HePC restored the altered metabolic status of infected macrophages, the impact of HePC being higher, confirming that disruption of mitochondrial functions is the primary leishmanicidal modality adopted by HePC (Fig. 7; Graphical abstract). This is perhaps the first study that has delineated the impact of conventional antileishmanials upon mitochondria-mediated metabolic bioenergetics in *L. donovani*-infected macrophages and can facilitate new drug discovery programs not only against *Leishmania* parasites but also against other trypanosomatids.

## Supplementary Information

Supplementary material 1: Fig. S1: Antileishmanial efficacy of conventional antileishmanials (Ampho B and HePC) in *Leishmania donovani *(*L. donovani*) promastigotes.

Supplementary material 2: Table S1: Antileishmanial efficacy of amphotericin B (Ampho B) and miltefosine (HePC) in *L. donovani* promastigotes and amastigotes. Table S2: Effect of conventional antileishmanials (acute response) on OCR and ECAR in *L. donovani* promastigotes.

Supplementary material 3: Fig. S2: Effect of acute treatment of conventional antileishmanials upon metabolic bioenergetics in *L. donovani* promastigotes.

Supplementary material 4: Figure legends of supplementary figures.

## Data Availability

Data generated during this study are included in this published article (and its supplementary information files).

## Abbreviations

AMPK: AMP-activated protein kinase
CaMKK-β: Calcium/calmodulin kinase kinase-β
CL: Cutaneous leishmaniasis
CPT1a: Carnitine palmitoyl transferase 1a
COX IV: Cytochrome c oxidase
ddPCR: Droplet digital polymerase chain reaction
ECAR: Extracellular acidification rate
ETC: Electron transport chain
FBS: Fetal bovine serum
FCCP: Trifluoromethoxyphenylhydrazone
GMFC: Geometrical mean fluorescence channel
H2DCFDA: 2,7-Dichlorodihydrofluoresceindiacetate
HKII: Hexokinase II
HePC: Hexadecylphosphocholine
H2O2: Hydrogen peroxide
LCFAs: Long-chain fatty acids
LDH: Lactate dehydrogenase
LKB1: Liver kinase B1
MCL: Muco-cutaneous leishmaniasis
MMP: Mitochondrial membrane potential
MPC: Mitochondrial pyruvate carrier
mTOR: Mammalian target of rapamycin
MTS: 3-(4,5-Dimethylthiazol-2-yl)-5-(3-carboxymethoxyphenyl)-2-(4-sulfonyl)-2*H*-tetrazolium
NEM: *N*-ethylmaleimide
NTDs: Neglected tropical diseases
OCR: Oxygen consumption rate
PCD: Programmed cell death
PDK1: Pyruvate dehydrogenase kinase 1
PFK: Phosphofructokinase
PGC1α: Peroxisome proliferator-activated receptor (PPAR)-γ coactivator 1α
PKM2: Pyruvate kinase M2
PMS: Phenazine methosulfate
SIRT1: Sirtuin1
TCA cycle: Tricarboxylic acid cycle
TV: ThiolTracker™ Violet
Rot + AA: Rotenone–antimycin A
VL: Visceral leishmaniasis
WHO: World Health Organization
