# Supplementary figures and images for: Targeting mitochondrial bioenergetics: the “Achilles’ heel” of Leishmania
Source: Parasit Vectors. 2026 Mar 9;19:165. doi: 10.1186/s13071-026-07247-x (PMC13085416; doi:10.1186/s13071-026-07247-x)

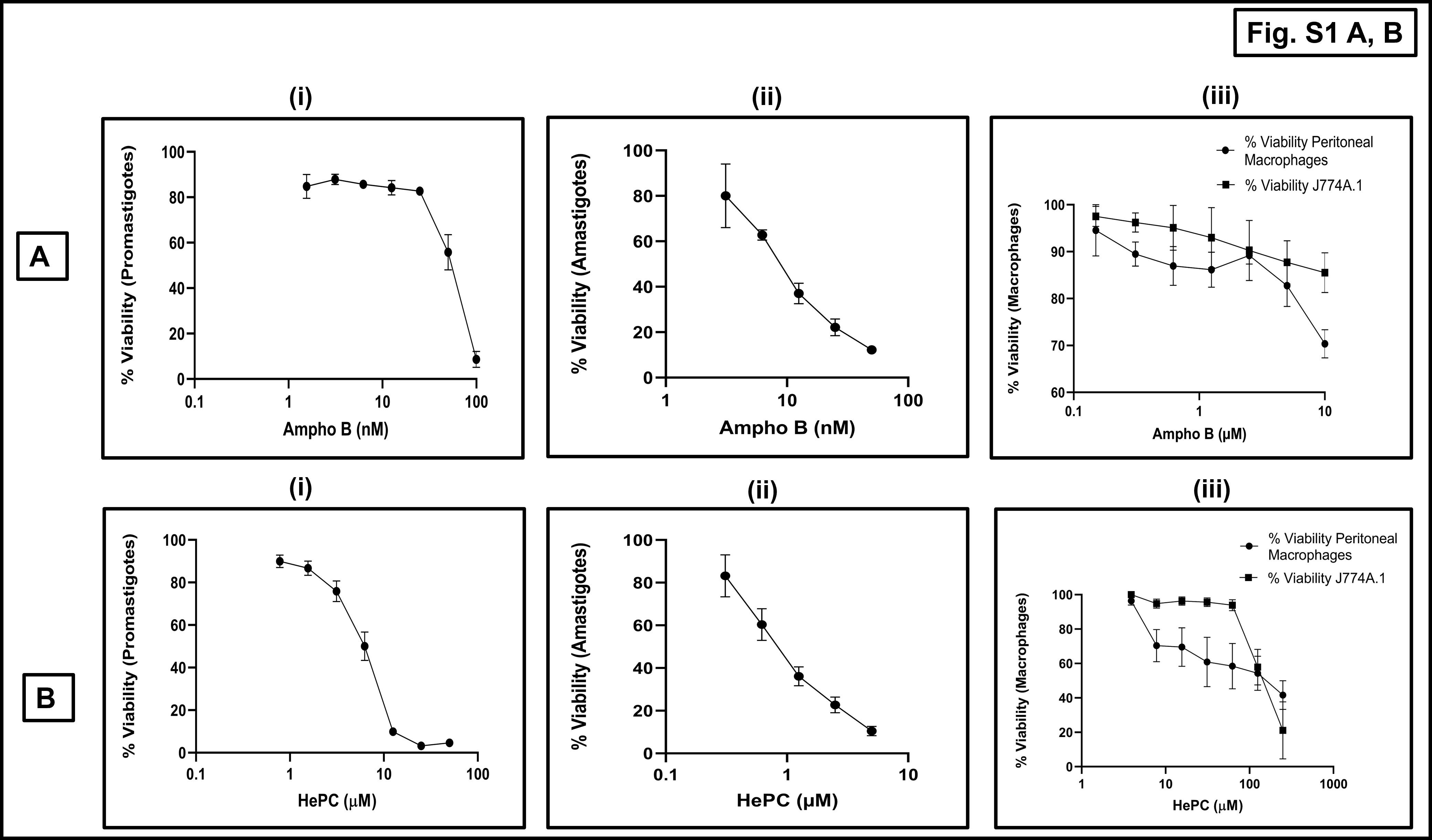

Supplement: Supplementary file 1 — Supplementary material 1: Fig. S1: Antileishmanial efficacy of conventional antileishmanials (Ampho B and HePC) in Leishmania donovani (L. donovani) promastigotes. [file 13071_2026_7247_MOESM1_ESM.tif]

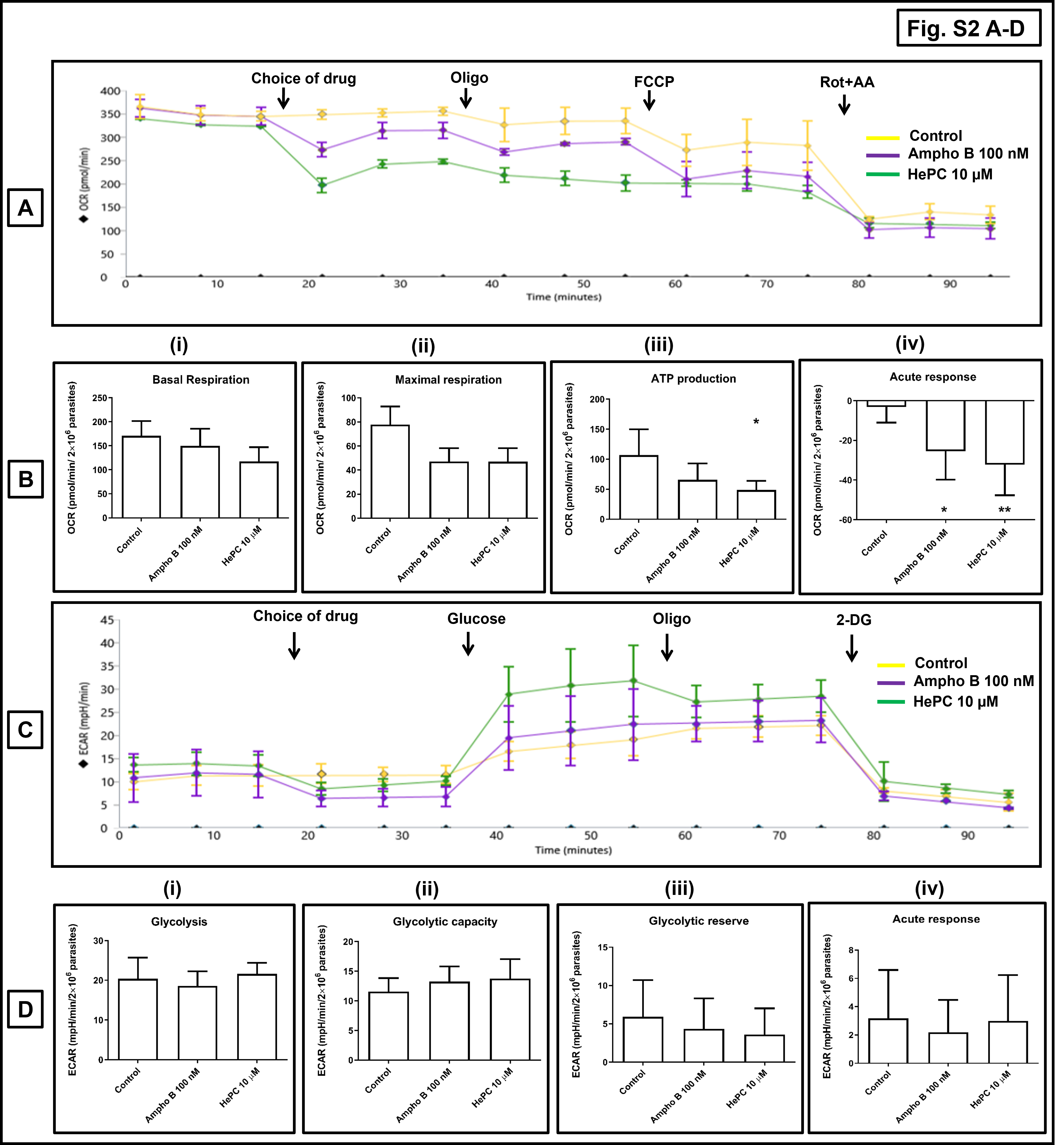

Supplement: Supplementary file 3 — Supplementary material 3: Fig. S2: Effect of acute treatment of conventional antileishmanials upon metabolic bioenergetics in L. donovani promastigotes. [file 13071_2026_7247_MOESM3_ESM.tif]
