# Supplementary material for: Targeting mitochondrial bioenergetics: the “Achilles’ heel” of Leishmania
Source: Parasit Vectors. 2026 Mar 9;19:165. doi: 10.1186/s13071-026-07247-x (PMC13085416; doi:10.1186/s13071-026-07247-x)
Supplement: Supplementary file 2 — Supplementary material 2: Table S1: Antileishmanial efficacy of amphotericin B (Ampho B) and miltefosine (HePC) in L. donovani promastigotes and amastigotes. Table S2: Effect of conventional antileishmanials (acute response) on OCR and ECAR in L. donovani promastigotes. [file 13071_2026_7247_MOESM2_ESM.docx]

**Supplementary information**

**Additional Tables**

**Table S1 Anti-leishmanial efficacy of Amphotericin B (Ampho B) and Miltefosine (HePC)**

| **Anti-leishmanial** | **Anti-promastigote**  **Activity** | **Anti-amastigote**  **Activity** | **Cytotoxicity in Macrophages** |
| --- | --- | --- | --- |
| Ampho B | IC_50_ = 52.30±04.05 nM  IC_90_ = 93.32±01.68 nM | IC_50_ = 09.42±0.38 nM  IC_90_ = 50.13± 0.76 nM | CC_50_ = ≥ 10 µM (both J774A.1 & peritoneal macrophages) |
| HePC | IC_50_ = 05.80±0.53 µM  IC_90_ = 11.66±0.66 µM | IC_50_ = 0.89±0.38 µM  IC_90_ = 04.73±0.51 µM | CC_50_=125.9±16.18 µM (J774.A1)  CC_50_=151.7±08.13µM (Peritoneal Macrophages) |

**A** Log phase promastigotes (AG83, 1×10^5^/200 µl/well) were incubated with Ampho B (0-100 nM) or HePC (0-25 µM). Cell viability was measured by the MTS-PMS assay, as described in Materials and methods and data are expressed as the mean ± SEM of at least three experiments in duplicates.

**B** AG83 infected murine peritoneal macrophages were incubated with Ampho B (0-50 nM) or HePC (0-5 µM) for 48 h and the anti-amastigote activity was determined by Giemsa staining as well as *A2* gene expression, as described in Materials and methods and data are expressed as the mean±SEM of at least three experiments in duplicates.

**C** J774A.1 and murine peritoneal macrophages were incubated with Ampho B (0-10 µM) or HePC (0-250 µM) for 48 h. Cell viability was measured by the MTS-PMS assay, as described in Materials and methods and data are expressed as the mean ± SEM of at least three experiments in duplicates.**Table S2A Effect of conventional anti-leishmanials (acute response) on mitochondrial oxygen consumption rate (OCR) in *L. donovani* promastigotes**

| **Parameters of**  **mitochondrial respiration**  **(pmol/min/2×10^6^ parasites)** | **Control** | **Ampho B**  **100 nM** | **HePC**  **10 µM** |
| --- | --- | --- | --- |
| Non-mitochondrial respiration | 80.51±15.28 | 74.81±13.11 | 76.55±15.86 |
| Basal respiration | 171.01±30.69 | 149.90±35.76 | 117.53±29.69 |
| Maximal respiration | 77.90±14.99 | 47.18±11.01 | 46.93±11.28 |
| Proton Leak | 96.85±26.31 | 63.80±19.23 | 47.91±14.33 |
| SRC (%) | 51.9±08.3 | 50.1±16.9 | 44.6±05.3 |
| Coupling efficiency (%) | 43.6±16.2 | 41.4±08.5 | 34.9±05.4 |
| ATP linked respiration | 106.8±42.92 | 65.52±27.25 | 48.60±15.34* |
| Acute response | -03.12±7.90 | -25.43±14.40* | -32.23±15.46** |

Mitochondrial respiration in terms of OCR of log phase AG83 promastigotes (2×10^6^/well) was measured by Seahorse Extracellular Flux Analyzer (XFp) following treatment with Ampho B (100 nM, acute response) and HePC (10 µM, acute response) by addition of these drugs in Port A followed by addition of oligomycin (Oligo 10 µM), FCCP (2 µM) and Rot+AA (1 µM, each), as described in Materials and methods. Data are expressed as mean±SEM of OCR (pmol/min/2×10^6^ parasites) of at least three experiments in duplicates; *p<0.05 and **p<0.01 as compared to control.

**Table S2B Effect of conventional anti-leishmanials (acute response) on extracellular acidification rate (ECAR) in *L. donovani* promastigotes**

| **Parameters of**  **glycolytic activities**  **(mpH/min/2×10^6^ parasites)** | **Control** | **Ampho B**  **100 nM** | **HePC**  **10 µM** |
| --- | --- | --- | --- |
| Non-glycolytic acidification | 11.58±02.24 | 10.72±02.28 | 12.77±02.30 |
| Glycolysis | 20.38±05.33 | 18.60±03.67 | 21.62±02.76 |
| Glycolytic capacity | 11.56±02.28 | 13.24±02.57 | 13.77±03.27 |
| Glycolytic reserve | 05.92±04.77 | 04.32±03.97 | 03.62±03.40 |
| Acute Response | 03.17±03.41 | 02.18±2.28 | 02.98±03.25 |

The effect of Ampho B (100 nM, acute response) and HePC (10 µM, acute response) upon the ECAR of log phase AG83 promastigotes (2×10^6^/well) was measured in a Seahorse Extracellular Flux Analyzer (XFp) by addition of these drugs in Port A followed by the addition of glucose (10 mM), oligomycin (Oligo 10 µM) and 2-DG (50 mM), as described in Materials and methods. Data are expressed as mean±SEM of ECAR (mpH/min/2×10^6^ parasites) of at least three experiments in duplicates.
