# Supplementary material for: Targeting mitochondrial bioenergetics: the “Achilles’ heel” of Leishmania
Source: Parasit Vectors. 2026 Mar 9;19:165. doi: 10.1186/s13071-026-07247-x (PMC13085416; doi:10.1186/s13071-026-07247-x)
Supplement: Supplementary file 4 — Supplementary material 4: Figure legends of supplementary figures. [file 13071_2026_7247_MOESM4_ESM.docx]

**Supplementary information**

**Additional Figure legends**

**Fig. S1 Anti-leishmanial efficacy of conventional anti-leishmanials (Ampho B and HePC) in *Leishmania donovani (L. donovani)***

**A, B (i)** Log phase promastigotes (AG83, 1×10^5^/200 µl/well) were incubated with Ampho B (0-100 nM) **(A)** or HePC (0-25 µM) **(B)**. Cell viability was measured by the MTS-PMS assay, as described in Materials and methods; data are expressed as the mean ± SEM of at least three experiments in duplicates.

**A, B (ii)** AG83 infected murine peritoneal macrophages were incubated with Ampho B (0-50 nM) **(A)** or HePC (0-5 µM) **(B)** for 48h and the anti-amastigote activity was determined by Giemsa staining as well as *A2* expression, as described in Materials and methods; data are expressed as the mean±SEM of at least three experiments in duplicates.

**A, B (iii)** J774A.1 (⏹) and murine peritoneal macrophages (⏺) were incubated with Ampho B (0-10 µM) **(A)** or HePC (0-250 µM) **(B)** for 48h. Cell viability was measured by the MTS-PMS assay, as described in Materials and methods; data are expressed as the mean ± SEM of at least three experiments in duplicates.

**Fig. S2** **Effect of acute treatment of conventional anti-leishmanials upon metabolic bioenergetics in *L. donovani* promastigotes**

**A** Representative profile of mitochondrial respiration (OCR) of at least three independent experiments of log phase promastigotes (AG83, 2×10^6^ cells/well, ) treated with Ampho B (100 nM, ) or HePC (10 µM, ) in Port A (acute response), followed by addition of oligomycin (Oligo 10 µM), FCCP (2 µM) and Rot+AA (1 µM, each), as measured by Seahorse Extracellular Flux Analyzer (XFp).

**B** Bar graphs of (i) basal respiration, (ii) maximal respiration, (iii) ATP production and (iv) acute response. Data is expressed as the mean±SEM of OCR (mpH/min/2×10^6^ parasites) of at least three experiments in duplicates; *p<0.05 and **p<0.01 as compared to untreated parasites.

**C** Representative profile of glycolytic activities (ECAR) of at least three different experiments of log phase promastigotes (AG83, 2×10^6^ cells/well, ) following treatment with Ampho B (100 nM, ) or HePC (10 µM, ) in Port A (acute response) and measured by Seahorse Extracellular Flux Analyzer (XFp) following addition of glucose (10 mM), oligomycin (Oligo 10 µM) and 2-DG (50 mM) as described in Materials and methods.

**D** Bar graphs of (i) glycolysis, (ii) glycolytic capacity, (iii) glycolytic reserve and (iv) acute response. Data is expressed as the mean±SEM of ECAR (mpH/min/2×10^6^ parasites) of at least three experiments in duplicates.
